# Soluble Epoxide Hydrolase Deletion Limits High-Fat Diet-Induced Inflammation

**DOI:** 10.3389/fphar.2021.778470

**Published:** 2021-12-17

**Authors:** Karen M. Wagner, Jun Yang, Christophe Morisseau, Bruce D. Hammock

**Affiliations:** Department of Entomology and Nematology, UC Davis Comprehensive Cancer Center, University of California Davis, Davis, CA, United States

**Keywords:** soluble epoxide hydrolase, epoxy-fatty acids (EpFA), omega-3, brown adipose tissue, eicosanoids

## Abstract

The soluble epoxide hydrolase (sEH) enzyme is a major regulator of bioactive lipids. The enzyme is highly expressed in liver and kidney and modulates levels of endogenous epoxy-fatty acids, which have pleiotropic biological effects including limiting inflammation, neuroinflammation, and hypertension. It has been hypothesized that inhibiting sEH has beneficial effects on limiting obesity and metabolic disease as well. There is a body of literature published on these effects, but typically only male subjects have been included. Here, we investigate the role of sEH in both male and female mice and use a global sEH knockout mouse model to compare the effects of diet and diet-induced obesity. The results demonstrate that sEH activity in the liver is modulated by high-fat diets more in male than in female mice. In addition, we characterized the sEH activity in high fat content tissues and demonstrated the influence of diet on levels of bioactive epoxy-fatty acids. The sEH KO animals had generally increased epoxy-fatty acids compared to wild-type mice but gained less body weight on higher-fat diets. Generally, proinflammatory prostaglandins and triglycerides were also lower in livers of sEH KO mice fed HFD. Thus, sEH activity, prostaglandins, and triglycerides increase in male mice on high-fat diet but are all limited by sEH ablation. Additionally, these changes also occur in female mice though at a different magnitude and are also improved by knockout of the sEH enzyme.

## 1 Introduction

The soluble epoxide hydrolase (sEH) enzyme plays a role in physiology and pathophysiology by regulating several classes of bioactive lipids, particularly epoxy-fatty acids (EpFA), which are rapidly hydrolyzed by the sEH. There have been several indications that this enzyme has a role in metabolic disease and obesity (Anandan et al., 2011; Luria et al., 2011; Overby et al., 2020). A high-fat diet (HFD) is known to alter the microbiome and GI tract cancers where sEH plays a role (Zhang et al., 2013b; Wang et al., 2017) and substitutions with omega 3 fatty acids can also have significant effects (Zhang et al., 2013a). The sEH has been observed to increase in inflammatory conditions (Ren et al., 2016), and HFD induces inflammation (Duan et al., 2018) and is also hypothesized to induce central nervous system (CNS) inflammation (Guillemot-Legris et al., 2016). Inhibiting the sEH with small-molecule inhibitors (sEHI) has been found to promote brown adipogenesis and reduce triglycerides in preclinical obesity (Overby et al., 2020). Here, we employ sEH ablation with the global gene knockout (sEH KO) to explore the role of sEH in different dietary conditions with both female and male mice. There is a demonstrated sexual dimorphism in sEH activity and expression (Gill and Hammock, 1980) (Pinot et al., 1995), and this may have biological consequences (Dewey et al., 2013; Vanella et al., 2015). Therefore, we investigated the role of sEH activity as it relates not only to differences in diet but also sex in preclinical species.

The sEH is the principal degradation path of epoxy-fatty acids (EpFA) formed from long chain polyunsaturated fatty acids (LC-PUFA) *via* cytochrome P450 activity. It has been previously determined that EpFA metabolites are the most dramatically altered lipid metabolites in adipose tissue from male mice (Wang et al., 2017). The epoxides of several classes of long chain fatty acids including linoleic acid (LA), arachidonic acid (ARA), docosahexaenoic acid (DHA), and alpha-linoleic acid (*α*-LA) were significantly altered in inguinal, gonadal adipose, and interscapular tissue in HFD male mice (Wang et al., 2017). Among the several studies of sEH in adipose tissue, the nomenclature is variable, and a clarification may be useful. Male rodents have been the most common sex used for experiments and therefore the gonadal white adipose tissue (WAT) is often described as the epididymal WAT. Because we include females in our studies, we identify these as gonadal WAT, which appears in some studies using males as well. Additionally, the inguinal white adipose tissue is subcutaneous rather than intraperitoneal fat and therefore takes either name. In our study, we chose to focus on the gonadal fat pad compared to other types of fat tissue and did not analyze inguinal WAT and therefore refer to the gonadal WAT by only the WAT acronym. Intrascapular fat is typically brown adipose tissue (BAT). There is on some occasion beige to white fat present, but in our samples, only the BAT was sampled. Thus, in our studies, we assessed WAT, BAT, brain, and liver, and these correspond to gonadal (epididymal) WAT and intrascapular adipose or iBAT in other studies.

Early investigations in male C57/B6 mice indicated that sEH expression in liver, kidney, and gonadal adipose as well as enzyme-specific activity did not alter between standard fat diet (SFD) and HFD fed animals (De Taeye et al., 2010). However, an increase in total activity (which may include other enzyme activity) from the gonadal WAT was observed in obese HFD mice. Another study demonstrated increased sEH activity in gonadal WAT of LDL receptor null mice fed an atherogenic diet (21% fat and 0.15% cholesterol) for 14 weeks measured using a fluorescent substrate assay (Shen et al., 2015). A caution is that the fluorescent assay used is sensitive to esterase activity and several other enzymes in addition to sEH (Jones et al., 2005). Unless the esterases are purified out or inhibited and glutathione depleted, other activities in tissues with this substrate are over 10-fold the sEH activity (Shihadih et al., 2020), leading to high risk of artifactual measurements. More recently, the lack of change in sEH expression in gonadal adipose in wild-type (WT) mice on HFD was corroborated (Wang et al., 2017). Additionally, a range of omega-3 ratio enriched diets (n3FD) maintaining fat content equal to standard chow did not alter sEH activity in murine liver from standard chow fed mice (Harris et al., 2016). The omega-3 enrichment has been proposed as an intervention for several disease-related pathologies including Alzheimer’s disease as well as cardiovascular disease (Shinto et al., 2014; Hu et al., 2019). It has been hypothesized that either lowering the omega-6 content in diet or enriching the omega-3 could have beneficial effects in these pathological conditions (Simopoulos, 2008). Moreover, a combination of approaches, both inhibiting sEH and omega-3 diet enrichment, is most likely to significantly reduce inflammation by decreasing inflammatory prostanoids and increasing inflammation resolving epoxides of omega-3 lipids such as EPA and DHA. Thus, while there was no previous report of sEH expression alteration in WT mice fed HFD up to 20 weeks of diet consumption, there have been notable changes in body weight, adipose fat pads, and oxylipins and therefore we investigated enzyme activity by the more sensitive and selective radiometric assay (Borhan et al., 1995) in several target tissues.

## 2 Materials and Methods

### 2.1 Animals

Experiments were conducted in accordance with the protocols approved by the Institutional Animal Care and Use Committee of the University of California. The soluble epoxide hydrolase KO mice on a C57/B6 background and the C57/B6 were maintained separately but housed in the same vivarium under identical conditions during the same time frame. Both soluble epoxide hydrolase KO mice and C57/B6 WT female and male mice (8 weeks old) were allowed to acclimate and then administered one of the following diets for 8 weeks: HFD (60 kcal% fat, purchased from Research Diet Inc., catalog number D12492), control diet (10 kcal% fat, D12450J from Research Diet Inc.) (http://www.researchdiets.com/opensourcediets/stock-diets/dio-series-diets), Omega-3 DHA enriched diet [Diet formulated by Research Diets, 15 kcal% fat, 6.25% of Solutex0365 (by weight)], or standard chow (Teklad Global 18% Protein Rodent Diet, 18 kcal % fat). The DHA diet was produced from Solutex oils with added t-BHQ and was vacuum packed under nitrogen and stored at −20°C. Female and male WT mice and sEH KO mice fed with all four diets were assessed; weight gain and chow consumption (per cage) were measured over the course of the study (Supplementary Figure S1). Tissues were sampled and immediately flash frozen at −80°C and later assessed for sEH activity liver triglycerides and oxylipin metabolites.

### 2.2 sEH Enzyme Activity

The enzymatic activity of sEH was measured using [^3^H]-*trans*-diphenylpropene oxide (*t*DPPO) as a substrate following published methods (Borhan et al., 1995).

### 2.3 Triglyceride and Total Cholesterol

Tissue total triglyceride and total cholesterol were measured at the National Mouse Metabolic Phenotyping Center (MMPC) at UC Davis per their published protocols found at (https://www.mmpc.org/shared/protocols.aspx).

### 2.4 LC-MS/MS Lipid Analysis

Tissue homogenates were extracted and analyzed for a focused metabolite profile per previously published methods (Yang et al., 2009; Yang et al., 2019). Briefly for the extraction, 10 μl of deuterated internal standard solution was added to the specified tissue samples and then 400 μl of cold methanol with 0.1% of acetic acid and 0.1% of butylate hydroxytoluene (BHT) was added to these tissue samples and stored at −80°C for 30 min. After freezing, samples were homogenized ball mills at 30 Hz for 10 min and then kept at −80°C overnight. The homogenates were centrifuged at 16,000 *g* for 10 min, the supernatants collected, and remaining pellets were washed with 100 μl of ice-cold methanol with 0.1% of acetic acid and 0.1% of BHT and centrifuged at 16,000 *g* for 10 min. The supernatants of each sample were combined and diluted with 2 ml of H_2_O and loaded onto Waters Oasis HLB 3cc (Waters, Milford, MA) solid phase extraction (SPE) cartridges. The final concentrations of internal standards are 100 nM, which were obtained by spiking 10 µl of 500 nM solutions before extraction.

The samples were measured on a 1200 SL ultra-high performance liquid chromatography (UHPLC) (Agilent, Santa Clara, CA) interfaced with a 4,000 QTRAP mass spectrometer (Sciex, Redwood City, CA). The separation conditions for the LC were optimized to separate critical pairs of lipid mediators, which share the same multiple reaction monitor (MRM) transitions. In brief, separation was achieved on an Agilent Eclipse Plus C18 150 × 2.1 mm 1.8 µm column with mobile phases of water with 0.1% of acetic acid as mobile phase A and acetonitrile/methanol (84/16) with 0.1% of acetic acid as mobile phase B. All the parameters on the mass spectrometer were optimized with pure standards (purchased from Cayman Chemical, Ann Arbor, MI) under negative mode. The schedule MRM scan mode was employed to increase the sensitivity of the measurement.

### 2.5 Data Analysis

Data were analyzed using SigmaPlot software (San Jose, CA). All data are expressed as the mean ± standard error of the mean (SEM). When data were normally distributed, statistical significance was determined using ANOVA; otherwise, significance was determined by Mann–Whitney *U* test. *p*-values less than 0.05 are reported as statistically significant.

## 3 Results

The expression and activity of sEH previously compared among select murine tissues demonstrates that activity follows a ratio of liver > kidney > WAT (epididymal) (De Taeye et al., 2010). We similarly observed relative ratios of high activity in the liver compared to adipose tissue. In these experiments, we also investigated BAT and observed a higher overall sEH activity level in BAT compared to WAT across diet groups present in both females and males.

### 3.1 sEH Activity

The sEH activity appeared to correlate with the different diet treatments across groups in WT mice. The low-fat diet (LFD, 10%) tended to have lower measured activity, particularly in WT females. The HFD (60%) group had the highest activity levels and demonstrated a sexual dimorphism with WT males having a more substantial increase in sEH activity. The n3FD tended to show the highest amount of variability in outcomes in all WT mice female and male but also demonstrated significant effects. We tested the sEH mice for both catalytic activity and the sEH protein to ensure that the line was a clear knockout. The line was originally derived on an S129 line (Sinal et al., 2000) but has since been backcrossed well over 10 generations on a C57/B6 background. These mice were tested for any residual activity and to confirm if there were any low levels of expression in the KO mice that could potentially be induced by the HFD. Thus, any changes in oxylipin profiles are unrelated to any modulated activity of any possible residual sEH message or protein in the KO mice.

**FIGURE 1:**
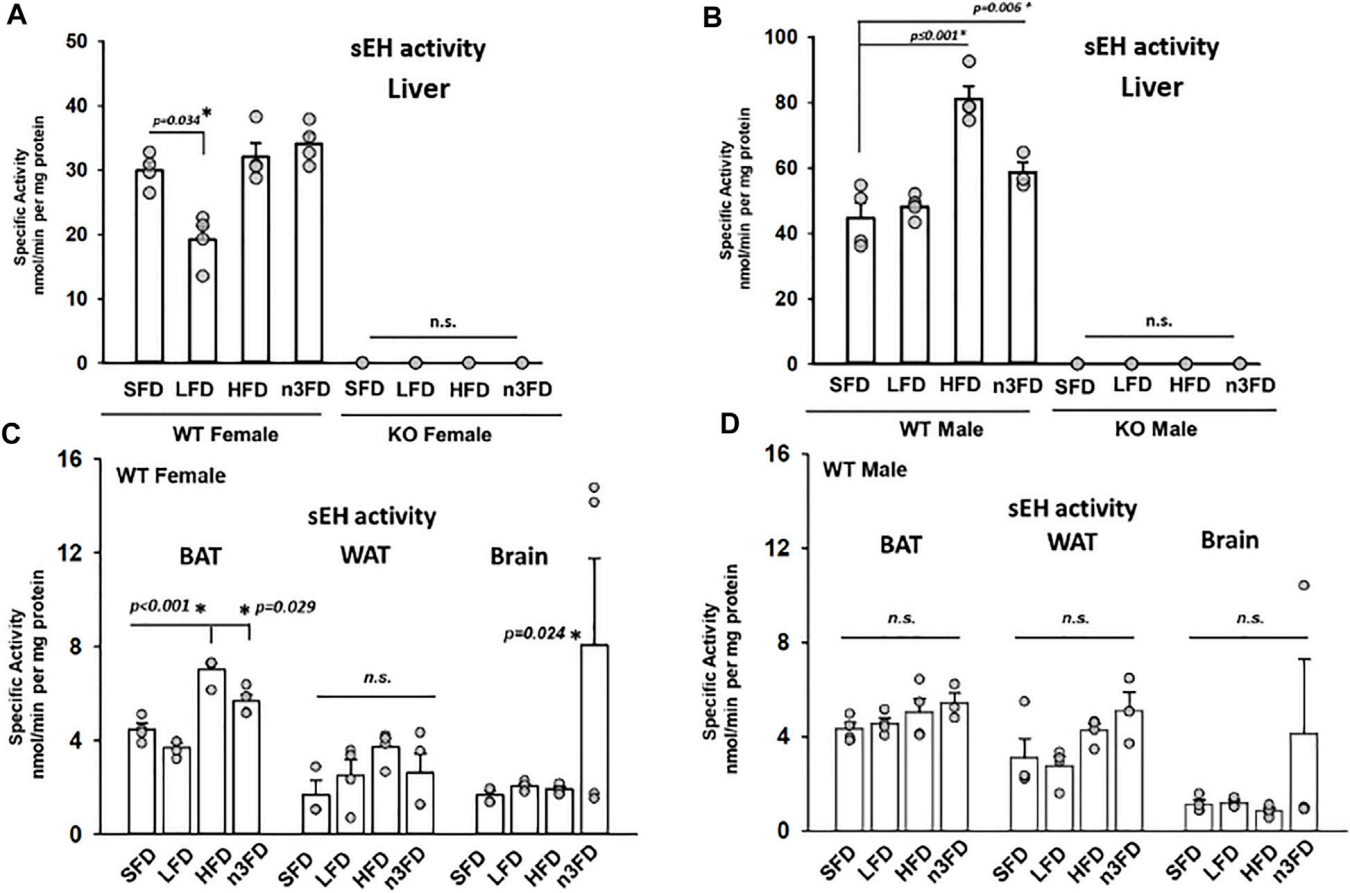
sEH activity in liver is responsive to dietary interventions. **(A)** In WT female liver, the most significant change was a decrease in activity on the LFD (10% total fat) diet. The sEH KO females were assessed on each of the diets to confirm their status, and as predicted, there was no enzyme activity (scale adjusted for <1 nmol per mg protein). **(B)** In WT male, the sEH activity responded to 8 weeks of diet with the HFD and n3FDs inducing increased activity in liver. sEH KO males like the KO females showed no activity in the assay. In other high fat content tissues, the sEH activity modulated in response to diet. The sEH KO females and males were assayed as controls and again showed no activity and are not depicted. **(C)** The WT female mice demonstrated increased activity in scapular brown adipose tissue (BAT) on the HFD and n3FD, which have increased fat content compared to LFD and SFD. The gonadal white adipose tissue (WAT) was not significantly altered by diets; however, the n3FD diet significantly increased sEH activity in the brain despite variability within the group. **(D)** WT male mice did not have significant change induced in the sEH activity per the different diet interventions in any of the three high fat content tissues investigated. Similar to the female WT mice, there was high variation within the n3FD group and this in males resulted in no significant change.

Measured with a sensitive radioactive substrate assay, the sEH activity in liver gave the most dynamic and statistically significant change (Figures 1A–D). Interestingly, in our experiments, the LFD significantly lowered the sEH activity in WT female liver compared to standard diet (Figure 1A, two-way ANOVA, Holm–Sidak post hoc, *p* = 0.034, *n* = 4/group). Note that for all these data on sEH activity, sEH activity is expressed in units of both mg/protein and mg/tissue in Supplementary Table S1. No activity was measurable in the sEH KO females (Figure 1A, two-way ANOVA, all groups male and female pairwise, *n* = 3–4/group, *p* = 0.687). There was also an increase in sEH activity in liver related to high dietary fat with the HFD in males (1B, two-way ANOVA, Holm–Sidak post hoc, *p* ≤ 0.001 HFD, *p* = 0.006 n3FD, *n* = 3–4/group) but not sEH KO males (Figure 1B). Overall, a significant difference was noted in activity in WT males compared to females across the different diets with males demonstrating higher sEH activity (one-way ANOVA, Dunn’s method post hoc, *p* ≤ 0.001, male vs. female, *n* = 15–16/sex). This result relates to previous data regarding the expression of the enzyme being higher in mature males compared to females (Gill and Hammock, 1980; Meijer et al., 1987). Interestingly, the sexual dimorphism in sEH activity in liver extended across all diets investigated in these experiments (two-way ANOVA, Holm–Sidak post hoc, *p* ≤ 0.001 male versus female, *n* = 3–4/group per sex).

In BAT, the HFD and n3FD increased the sEH activity in females (Figure 1C) (one-way ANOVA, Dunnett’s post hoc compared to SFD, *p* ≤ 0.001 HFD, *p* = 0.029 n3FD, *n* = 4/group). However, there was no difference in diet treatment on sEH activity in males (Figure 1D, one-way ANOVA, Dunnett’s post hoc *p* = 0.277, *n* = 3–4/group). Like the results in liver, there was no activity in the sEH KO animals, female or male (not depicted). Although the sexual dimorphism did not result in significant changes in lower-fat diets, the HFD increased the sEH activity in WT females compared to males (two-way ANOVA, Holm–Sidak post hoc, *p* ≤ 0.001 female versus male, *n* = 3–4/group per sex).

In the gonadal WAT, there was no statistical difference in activity between the diets for females or males [one-way ANOVA compared to SD, *n* = 3–4/group both sexes, *p* = 0.239 females (1C), *p* = 0.071 males (1D)]. However, the sexual dimorphism in WT she activity in WAT was significant (two-way ANOVA, Holm–Sidak post hoc, *p* = 0.014 male versus female, *n* = 3–4/group per sex).

Notably, in brain, sEH activity for both female and male WT groups showed a large population divergence on the n3FD and therefore no statistically significant changes between the sexes when compared with one-way ANOVA. This was an unanticipated amount of variability observed in the n3FD groups and speaks to the possibility of subpopulations in responding to the dietary changes that warrant further investigation. However, despite this variability, the n3FD diet did statistically increase the sEH activity in the WT female brain (Figure 1C, two-way ANOVA Holm–Sidak method post hoc, *p* = 0.024 n3FD vs. SD in females, *n* = 3–4/group). The sEH KO females and males showed no activity as expected (not depicted).

### 3.2 Body Weight

**FIGURE 2:**
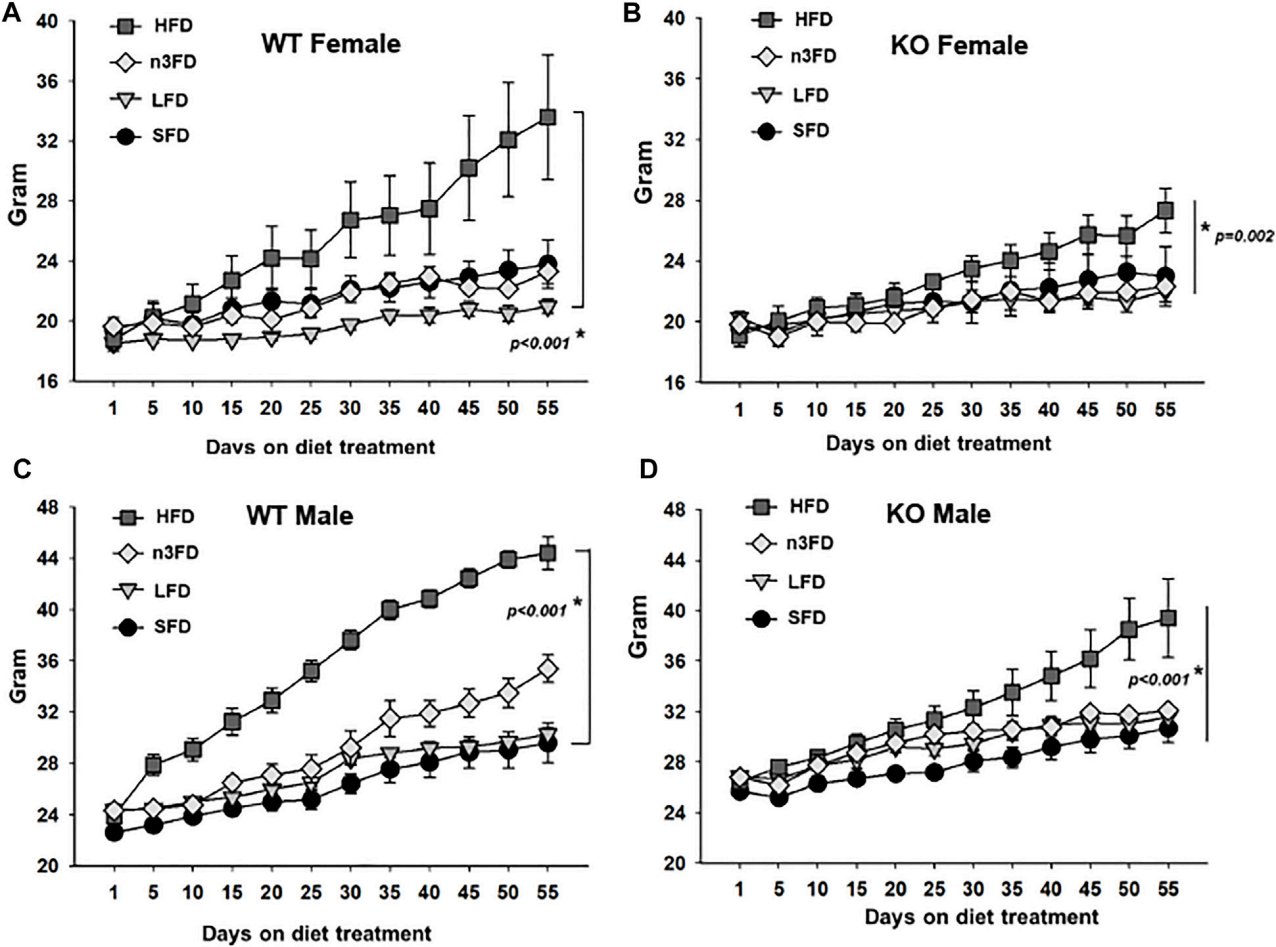
sEH KO animals show less change than WT mice after 8 weeks of diet treatment. **(A)** WT females responded to the HFD with increased weight gain and also increased variability in weight, which was still significant to the LFD. **(B)** The sEH KO females also demonstrated a significant increase in body weight on the HFD; however, this was less than the change seen in WT mice. **(C)** WT males responded significantly to the HFD and understandably demonstrated a sexual dimorphism gaining more than WT females. They also outpaced the increase in body weight compared to sEH KO male mice. **(D)** The sEH KO males also responded to the HFD with significant body weight increases and were also increased over sEH KO females. Whole body weight for all animals is shown in grams.

Notably, the body weights demonstrated the same change as the sEH activity in the WT female mice (Figure 2A). The weight of LFD WT female mice was significantly lower compared to the HFD after the 8-week treatment (two-way repeated measures ANOVA, Holm–Sidak post hoc, *p* < 0.028, *n* = 4/group). By day 45 and beyond, the HFD fed WT female mice had significantly increased body weight compared to all other diet groups (two-way repeated measures ANOVA, Holm–Sidak post hoc, *p* < 0.001, *n* = 4/group). The female sEH KO responded as well with the HFD increasing body weight significantly over the other three diet groups (Figure 2B, two-way repeated measures ANOVA, Holm–Sidak post hoc, *p* < 0.002–0.010 HDF vs. other diets *n* = 3/group). The body weight of WT male mice demonstrated the most significant increase on the HDF compared to the SFD treated for 8 weeks (Figure 2C). Despite the increased variability with the n3FD, WT males also significantly increased on n3FD compared to SFD (two-way repeated measures ANOVA, Holm–Sidak post hoc, *p* < 0.001, *n* = 4/group). Understandably, the weight of the male WT mice increased significantly compared to the WT females over time (two-way repeated measures ANOVA, Holm–Sidak post hoc, *p* < 0.001, *n* = 4/group).

The male sEH KO mice also responded to the 8 weeks of diet with the males showing significant increases in the HFD compared to SFD, LFD, and n3FD diets (Figure 2D, two-way repeated measures ANOVA, Holm–Sidak post hoc, *p* < 0.001–0.006 HDF 60% vs. other diets *n* = 3–4/group). The n3FD also increased male body weight compared to the SFD (*p* = 0.025). Similar to the WT mice, the male sEH KO mice increased in weight significantly compared to the females in all diets (two-way repeated measures ANOVA, Holm–Sidak post hoc, *p* < 0.001, *n* = 3–4/group).

When comparing the WT to sEH KO male mice, the WT males demonstrated a significant increase in body weight on the HFD [two-way repeated measures ANOVA, Holm–Sidak post hoc, *p* = 0.011 (*p* < 0.001 at Day 55), *n* = 3–4/group]. There were no significant differences when comparing WT female to sEH KO female mice on any diet (two-way repeated measures ANOVA, Holm–Sidak post hoc, *p* = 0.071–963, *n* = 3–4/group). Thus, the sexual dimorphism in body weight was the strongest relationship comparing all groups; however, significant increases with HFD in WT males compared to KO male mice demonstrated specifically the effect of sEH gene deletion.

### 3.3 Liver Triglyceride and Total Cholesterol

**FIGURE 3:**
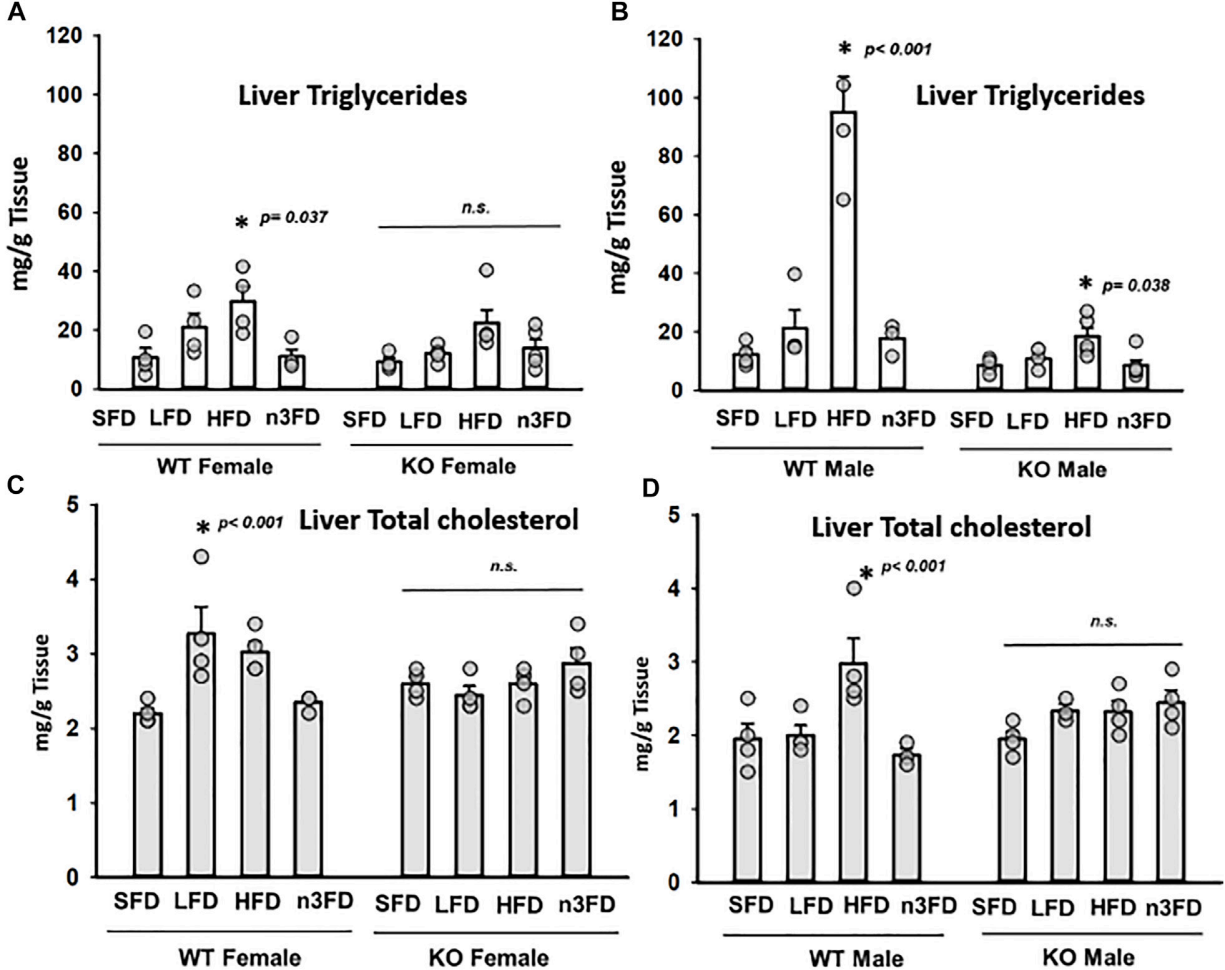
Total triglyceride (TG) and total cholesterol (TC) levels in liver. **(A)** In WT females, the HFD significantly increased TG levels in liver. The sEH KO female mice demonstrated no significant change. In general, increased dietary fat content increased TG levels in both WT and sEH KO females; however, the n3FD-treated mice not responding despite the increased fat content compared to SFD and LFDs is noteworthy. **(B)** The WT males showed the greatest response to the HFD with increased TG levels. However, the sEH KO males also had a significant response to the HFD. Interestingly for TG levels, there was not a large sexual dimorphic difference in WT or sEH KO mice. **(C)** Total cholesterol did alter with increasing fat content in the diet in WT females although there was an increase on the LFD. The TC level appeared to increase on the n3FD in sEH KO females but did not reach statistical significance. **(D)** The WT males responded to the HFD with higher TC levels but the sEH KO males similar to the sEH KO females did not respond but tended to increase on the n3FD.

In the WT females, the HFD increased triglycerides (TG) significantly compared to the SFD and n3FD (Figure 3A, one-way ANOVA, Holm–Sidak post hoc, *p* ≤ 0.037, *n* = 4/group). Interestingly, the diets did not induce any change in sEH KO females (3A, Kruskal–Wallis one-way ANOVA on Ranks, H = 7.777 with 3 degrees of freedom, *p* = 0.051, *n* = 4/group). In WT males, the liver TG increased with the HFD compared to all other diets (Figure 3B, one-way ANOVA, Holm Sidak post hoc, *p* < 0.001, *n* = 4/group). However, like the WT males, the sEH KO males responded to the HFD with significantly increased triglycerides compared to the SFD and n3FD (3B, one-way ANOVA, Holm–Sidak post hoc, *p* ≤ 0.038, *n* = 3–4/group). Comparing the male to female TGs, WT males were significantly higher than WT females, but the sEH KO males were not significantly increased compared to the KO female groups (two-way ANOVA, Holm–Sidak post hoc, *p* < 0.001 WT, *p* = 0.246 sEH KO, *n* = 3–4/group). Importantly the KO males had significantly lower TG across diet groups compared to WT males (Kruskal–Wallis one-way ANOVA on Ranks, Dunn’s post hoc, *p* = 0.002, *n* = 16–18/gene group). The female WT versus KO comparison was not significantly different with the same analysis (*p* = 0.380).

Total cholesterol (TC) in WT females varied from the TG results with the LFD demonstrating a significant increase compared to SFD (Figure 3C, Kruskal–Wallis one-way ANOVA on Ranks, *p* = 0.029, *n* = 4/group). Female KO lacked significant difference when compared across diets (3A, one-way ANOVA, Holm–Sidak post hoc, *p* = 0.423, *n* = 3–4/group). Similar to the TG results, the liver TC increased with the HFD in WT males compared to all diets (Figure 3D, one-way ANOVA, Holm Sidak post hoc, *p* < 0.043, *n* = 4/group). However, the TC levels in KO male mice lacked significant difference when compared across diets (3D, one-way ANOVA, Holm–Sidak post hoc, *p* = 0.054, *n* = 3–4/group). Comparing the TC in WT to sEH KO male mice revealed significant differences in the HFD (*p* = 0.005) and n3FD groups (*p* = 0.012) (two-way ANOVA, Holm–Sidak post hoc, *n* = 3–4/group). In WT females, the LFD (*p* = 0.006) and n3FD (*p* = 0.014) demonstrated significant increases in TC versus female KO (two-way ANOVA, Holm–Sidak post hoc, *n* = 4/group). WT females on the LFD also had higher TC than males (two-way ANOVA, Holm–Sidak post hoc, *p* < 0.001, *n* = 4/group). In the KO mice, the SFD and n3FD demonstrated differences in TC with males lower than females (two-way ANOVA, Holm–Sidak post hoc, *p* = 0.009 SFD, *p* = 0.007 n3FD, *n* = 4/group).

### 3.4 Oxylipins

#### 3.4.1 EpFA

**FIGURE 4:**
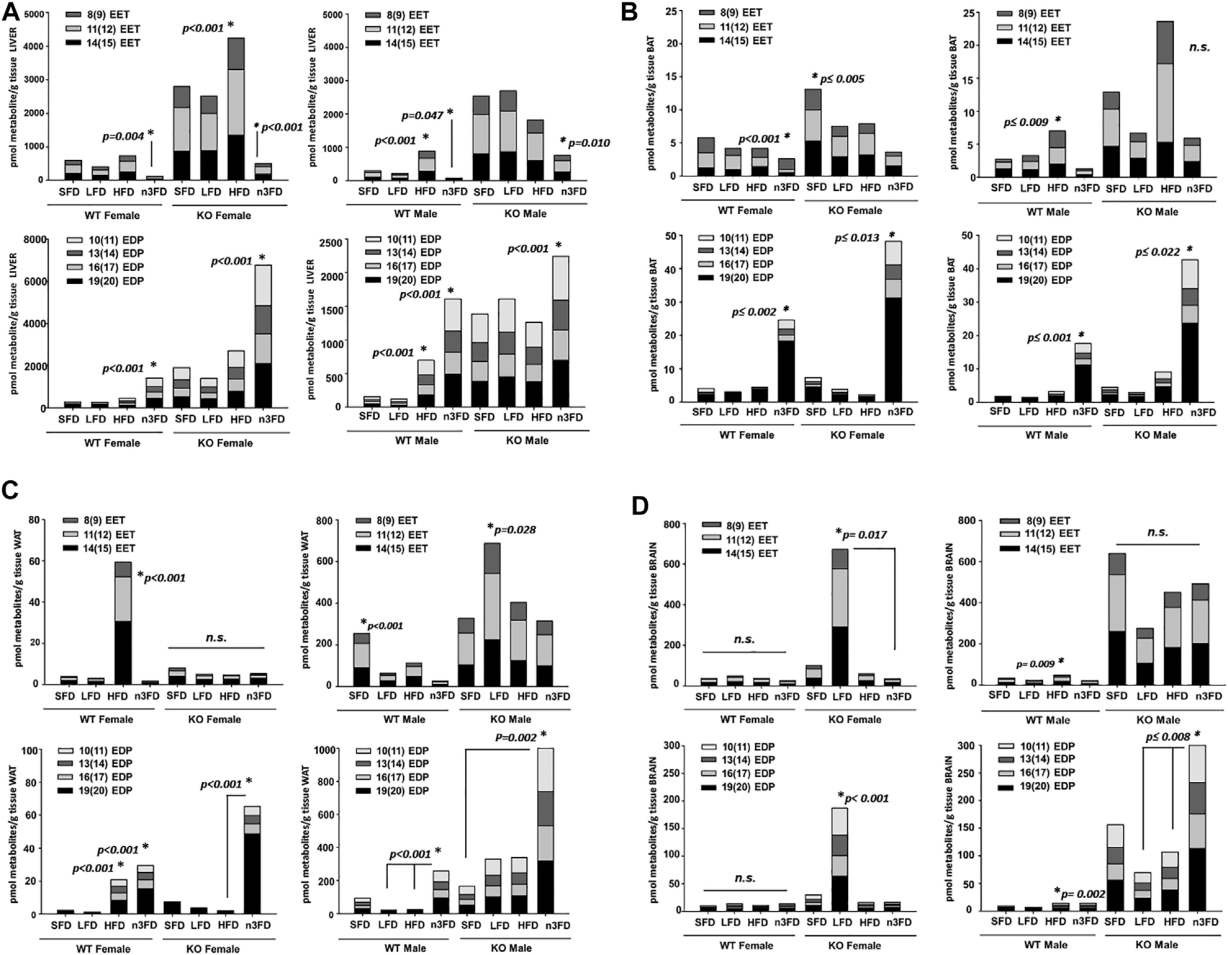
Oxylipins respond class of fat diet and strongly to genotype. **(A)** Liver: Selected epoxy-fatty acids (EpFA) derived from arachidonic acid (EETs) and docosahexaenoic acid (EDPs) were analyzed for change due to dietary intervention and genotype. In liver, the EET levels in female WT mice were significantly decreased on the n3FD **(top left)**. This also occurred in the sEH KO females, which had decreased EETs on the n3FD but also increased EETs on the HFD. In WT males, the EETs increased on the HFD and decreased on the n3FD **(top right)**. In the sEH KO males, the n3FD decreased the EETs, therefore resulting in all groups decreasing in EETs on the n3FD. Then, assessing EDPs in liver, the n3FD significantly increased levels in WT females **(bottom left)**. This was also true for the sEH KO females where the significant increase in EDPs was several-fold increased compared to the WT. In WT male, the HFD as well as the n3FD significantly increased the EDP levels **(bottom right)**. The sEH KO males interestingly did not show an increase on the HFD but like the other groups had a significantly increased level of EDPs in the n3FD. **(B)** BAT: EETs decreased in n3FD-treated WT females in BAT **(top left)**. The sEH KO females had increased levels of EETs in BAT on the SFD compared to all other diets with the lowest level measured in the n3FD fed animals. The WT males, as with other tissues, had increased levels of EETs on the HFD and also low levels on the n3FD **(top right)**. In WT females, the EDP levels were significantly increased on the n3FD which also was the result in the sEH KO females **(bottom left)**. The WT males and sEH KO males both showed increases in EDP levels on the n3FD **(bottom right)**. It is important to note that although the amount of change within the BAT analysis in these cases was dynamic, the absolute amount of oxylipins in this tissue were comparatively low. **(C)** WAT: The WAT of WT females was impacted by the HFD with significant increases in EETs but little change in this class of oxylipin was seen on other diets **(top left)**. The sEH KO females displayed no change among diets. Notably, the absolute amount of oxylipins for all groups were low in female WAT compared to liver regardless of genotype. The WT males had increased levels of EETs on the SFD and also low levels on the n3FD **(top right)**. The sEH KO males had higher levels compared to WT males and significantly higher EETs on the LFD. The EDP metabolites were also significantly higher on the HFD in WT females, but these metabolites were also higher in mice on the n3FD **(bottom left)**. The sEH KO females demonstrated a greater amount of change in the EDPs, which were significantly increased on the n3FD. The sEH KO males were increased on the n3FD in WAT (notice scale, **bottom right**). The results of this tissue demonstrated a genotypic difference that was dependent on the sexual dimorphism resulting in higher oxylipins of both classes in male sEH KO animals. **(D)** Brain: The WT female had no significant change in brain EETs, and the sEH KO female mice demonstrated a large increase only in mice on the LFD. In the WT male the EETs increased significantly but less dramatically on the HFD **(top left)** and sEH KO males, despite the appearance in increase in SFD and HFD groups, had no significant change **(top right)**. The changes in EDP levels were not significant in WT females and sEH KO females again showed increase only on LFD **(bottom left)**. Increased EDPs were observed for male WT on HFD and for sEH KO mice on the n3FD **(bottom right)**.

In liver (Figure 4A), the n3FD significantly reduced the EETs of all three regioisomers in female WT mice (two-way ANOVA, Holm–Sidak post hoc, *p* = 0.004 female, *n* = 4/group). In KO females, the n3FD significantly lowered the liver concentrations of EET regioisomers and the HFD increased them (two-way ANOVA, Holm–Sidak post hoc, *p* < 0.001 n3FD and HFD, *n* = 3–4/group). WT males, like the WT females, had reduced EETs on the n3FD (two-way ANOVA, Holm–Sidak post hoc, *p* = 0.047 male, *n* = 4/group). KO males on the n3FD also decreased the EET regioisomers compared to the SFD and LFD (two-way ANOVA, Holm–Sidak post hoc, *p* = 0.010 SFD and LFD, *n* = 3–4/group). This is remarkable because the n3FD retains the 6.2% corn oil and has more fat content (15% total) than the LFD (10%). This is consistent with the ω-olefin of EPA and DHA being excellent substrates for cytochromes P450s that oxidize polyunsaturated lipids (Arnold et al., 2010).

The liver EDPs increased in WT females on the n3FD compared all other diets (two-way ANOVA, Holm–Sidak post hoc, *p* < 0.001, *n* = 3–4/group). The same increases of EDPs occurred in the sEH KO females on the n3FD (two-way ANOVA, Holm–Sidak post hoc, *p* < 0.001, *n* = 3–4/group). The WT males on the n3FD had higher EDP levels compared to all other diets. The HFD in WT males also increased the EDPs compared to the SFD and LFD (two-way ANOVA, Holm–Sidak post hoc, *p* < 0.001, *n* = 3–4/group in each case). In KO males the EDPs increased on the n3FD compared to the SFD and HFD (two-way ANOVA, Holm–Sidak post hoc, *p* ≤ 0.032, *n* = 3–4/group). What is remarkable about the result is the difference in scale of EDPs in the liver between the female KO on the n3FD and the males. The total amount of EDPs in the female KO liver was 3-fold higher than the KO males while the WT were not significantly different between sexes (two-way ANOVA, Holm–Sidak post hoc, *p* < 0.001, *n* = 3–4/group). The KO females had increased EDPs on the n3FD and SFD compared to the corresponding WT of same sex and same diet (two-way ANOVA, Holm–Sidak post hoc, *p* < 0.001, *n* = 3–4/group). The KO males had increased EDPs on all diets different from WT males (two-way ANOVA, Holm–Sidak post hoc, *p* ≤ 0.048, *n* = 3–4/group).

EETs in BAT (Figure 4B) significantly decreased on the n3FD in WT female (Kruskal–Wallis one-way ANOVA on Ranks, n3FD vs. HFD, *p* < 0.001, *n* = 3–4/group). KO females were higher than WT females overall and had the highest EET levels on the SFD (one-way ANOVA on Ranks, Holm–Sidak post hoc, SFD vs. LFD, HFD, n3FD, *p* ≤ 0.005, *n* = 3–4/group). WT male demonstrated increases in EETs due to the HFD (Kruskal–Wallis one-way ANOVA on Ranks, HFD vs. SFD, n3FD, *p* ≤ 0.009, *n* = 3–4/group). Surprisingly, the KO males showed increases in EETs but were not significant although they showed significant increases with HFD (Kruskal–Wallis one-way ANOVA on Ranks, Dunn’s post hoc, *p* = 0.072, *n* = 3–4/group).

EDPs in WT females predictably increased on the n3FD (Kruskal–Wallis one-way ANOVA on Ranks, n3FD vs. SFD, HFD, n3FD, *p* ≤ 0.002, *n* = 3–4/group) which was true for the KO female as well (Kruskal–Wallis one-way ANOVA on Ranks, Dunn’s post hoc, n3FD vs. SFD, LFD, HFD, *p* ≤ 0.019, *n* = 3–4/group). The WT male (Kruskal–Wallis one-way ANOVA on Ranks, Dunn’s post hoc, n3FD vs. SFD, LFD, *p* ≤ 0.001, *n* = 3–4/group) and sEH KO male both had higher EDPs on the n3FD as well (Kruskal–Wallis one-way ANOVA on Ranks, Dunn’s post hoc, n3FD vs. LFD, *p* ≤ 0.022, *n* = 3–4/group).

In WAT (Figure 4C), the EETs of all three included regioisomers increased in WT female on HFD (Kruskal–Wallis one-way ANOVA on Ranks, HFD vs. n3FD, LFD, *p* ≤ 0.001, SFD, *p* = 0.003, *n* = 4/group). KO female had no significant change related to diet (*p* = 0.153). WT male had the highest levels on SFD (Kruskal–Wallis one-way ANOVA on Ranks, SFD vs. n3FD, LFD, *p* ≤ 0.031, HFD vs. n3FD, *p* = 0.007, *n* = 4/group). The KO male EETs were higher than all other groups and had significantly higher levels on the LFD (Kruskal–Wallis one-way ANOVA on Ranks. SFD vs. LFD, *p* = 0.028).

The WAT EDPs were significantly higher on both the HFD and n3FD in WT female (Kruskal–Wallis one-way ANOVA on Ranks, Dunn’s post hoc, n3FD vs. SFD and LFD, *p* ≤ 0.001, HFD vs. SFD and LFD, *p* ≤ 0.001, *n* = 4/group). In the KO female, the n3FD raised EDPs (Kruskal–Wallis one-way ANOVA on Ranks, Dunn’s post hoc, n3FD vs. HFD, *p* < 0.001, vs. LFD and SFD, *p* ≤ 0.013, *n* = 3–4/group). The WT male also had elevated EDPs on the n3FD (Kruskal–Wallis one-way ANOVA on Ranks, Dunn’s post hoc, n3FD vs. HFD, LFD, *p* ≤ 0.001, *n* = 4/group). However, the KO males had the largest increase in EDPs overall (Kruskal–Wallis one-way ANOVA on Ranks, Dunn’s post hoc, n3FD vs. SFD, *p* = 0.002, *n* = 3–4/group).

In the brain (Figure 4D), EET levels in the WT females were not altered by the different diets (Kruskal–Wallis one-way ANOVA on Ranks, *p* = 0.104, *n* = 4/group). The female KO mice responded with a seemingly large increase in EETs on the LFD, which was significant compared to only the n3FD (Kruskal–Wallis one-way ANOVA on Ranks, Dunn’s post hoc, n3FD vs. LFD, *p* ≤ 0.017, *n* = 3–4/group). The WT male EETs increased on the HFD compared to both the n3FD and LFD, but overall, the levels were compared to the KO males (Kruskal–Wallis one-way ANOVA on Ranks, Dunn’s post hoc, n3FD vs. LFD, *p* ≤ 0.009, *n* = 3–4/group). The changes in male sEH KO EETs were not significant among the diet groups (*p* = 0.227 in same analysis) likely due to variability and the small *n* per group. However, these levels were nonetheless much higher than levels measured in WT animals.

The WT female EDPs did not change with the different diets (*p* = 0.132). The KO females, similar to the EETs results, had a large increase in EDPs on the LFD compared to other diets (two-way ANOVA, Holm–Sidak post hoc, *p* < 0.001, *n* = 3–4/group). The WT male EDPs increased in the HFD compared to both the n3FD and LFD (Kruskal–Wallis one-way ANOVA on Ranks, Dunn’s post hoc, n3FD vs. LFD, *p* ≤ 0.002, *n* = 3–4/group). However, again the change in EDPs for WT males was still folds lower than levels in the sEH KO males. In the sEH KO males, EDPs increased in response to the n3FD compared to both the LFD and HFD (Kruskal–Wallis one-way ANOVA on Ranks, Dunn’s post hoc, n3FD vs. LFD, *p* ≤ 0.008, *n* = 3–4/group) and the levels overall were much higher that WT animals and the KO females with the exception of the LFD effect in KO female. Numerical values for the averages ± SEM for individual regioisomers of the EpFA analyzed here are also reported in Supplementary Table S2.

#### 3.4.2 Prostaglandins

**FIGURE 5:**
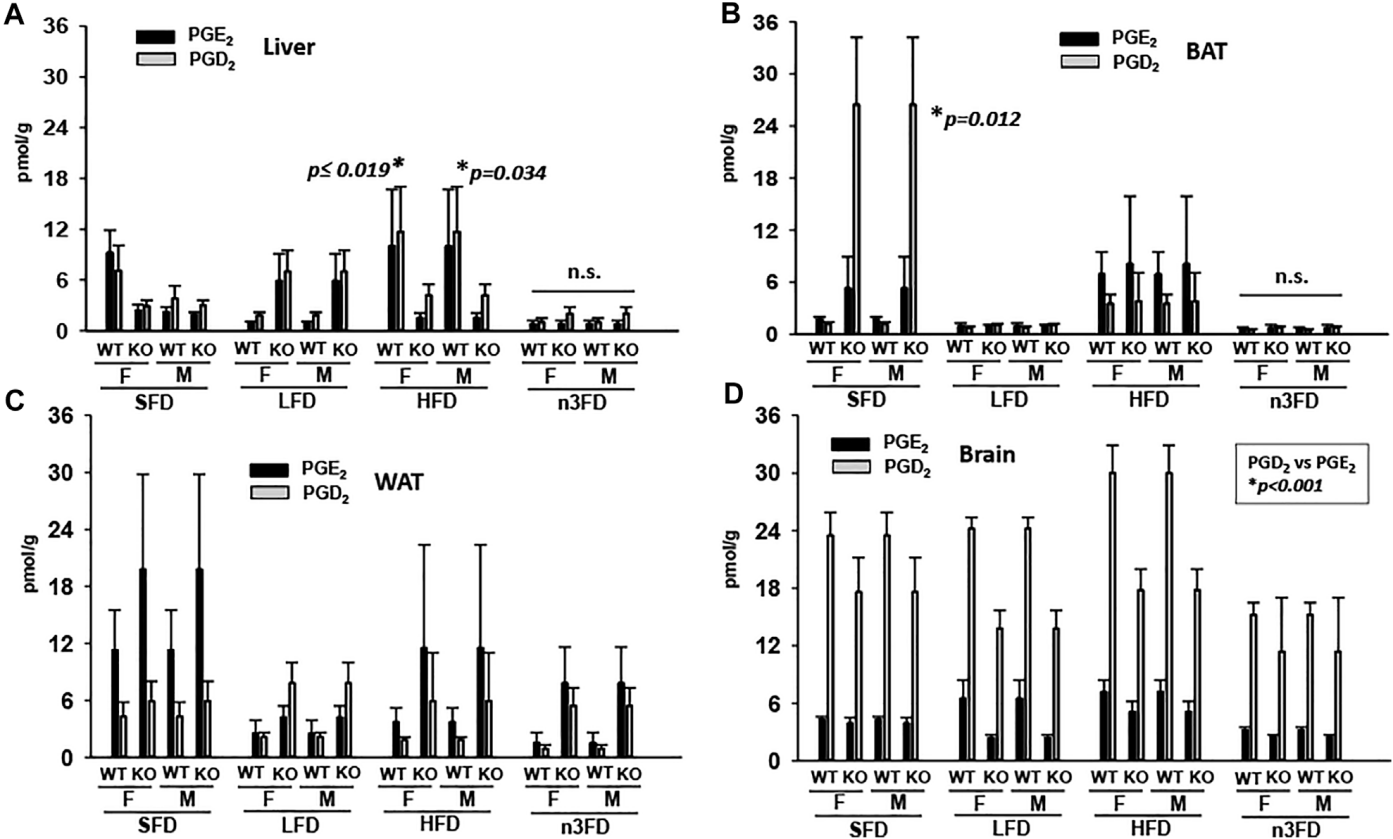
Proinflammatory prostaglandins increase with HFD. **(A)** In liver PGE_2_ and PGD_2_ increase on HFD in mice of both sexes and genotype. **(B)** This is also the case in the BAT for mice with the notable difference that LFD and n3FD demonstrated decreased prostaglandins. **(C)** The WAT demonstrated the highest levels of prostaglandins, even on the SFD and these were lowered in LFD and n3FD especially in WT animals. **(D)** There was minimal change related to prostaglandins in the brain due to diet; however, large differences between the prostaglandin levels were observed.

The depicted prostaglandins are recognized as two of the most proinflammatory endogenous lipid metabolites. In the liver of WT mice, both female and male PGE_2_ and PGD_2_ increased on the HFD and decreased on the n3FD (Figure 5A, Kruskal–Wallis one-way ANOVA on Ranks, *p* ≤ 0.019, *n* = 4/group female, *p* = 0.034, *n* = 4/group male). This was not the case in KO mice where prostaglandins in both female and male increased on the LFD compared to WT, though the KO mice also had lower levels in the n3FD. Overall, these prostaglandins were low in BAT especially on the LFD and n3FD in all mice. However, the SFD displayed increases in PGD2 levels in KO mice (Figure 5B, two-way ANOVA on Ranks, Holm–Sidak post hoc, *p* = 0.012, *n* = 3–4/group). The inflammatory prostaglandins increased in BAT with HFD in all mice compared to LFD and n3FD. The WAT (Figure 5C), showed the highest levels of the prostaglandins, which tended to be higher in sEH KO mice compared to WT mice but did not reach significance. In the brain (Figure 5D), there were slight decreases in PGE_2_ in the sEH KO mice compared to WT on LFD and HFD. PGD_2_ is known to be a more abundant prostaglandin in brain and understandably demonstrated much higher levels than PGE_2_ (two-way ANOVA on Ranks, Holm–Sidak post hoc, PGD_2_ vs. PGE_2_ *p* < 0.001, *n* = 3–4/group). For numerical values for the averages ±SEM for these prostaglandins from cyclooxygenase metabolism, see Supplementary Table S2.

## 4 Discussion

Several studies have laid the groundwork for the role of sEH in obesity. Protein expression levels of the phospholipases that liberate PUFAs from cellular membranes, the CYP enzymes that generate the EpFA substrates of sEH, sEH itself, and other enzymes in arms of the ARA cascade have been measured and correlated with inflammatory markers. These experiments have provided the information that sEH has a role in modulating the regulation of EpFA in obesity to improve health. The current study has furthered this demonstration, moving beyond the expression to the activity of sEH as a lipid-regulating enzyme in tissues with a high lipid content. An important outcome of these experiments is the demonstration of relative sEH activity in the adipose tissues compared to the brain and liver. Importantly, it also addresses the differences for female compared to male animals in all the included analyses. The liver remains the tissue with the highest sEH expression and activity, and the liver may play a role in physiopathology including in CNS diseases such as Alzheimer’s disease (Estrada et al., 2019). This disease, in particular, has also demonstrated a sexual dimorphism (Fisher et al., 2018; Zhu et al., 2021) and an examination of lipid metabolism may yield information useful to determine the physiological consequences of imbalances that may coincide with or indicate susceptibility to disease states.

It is important to note when interpreting the results that few of the several studies investigating the role of sEH with dietary interventions follow identical experimental design. Some studies have used HFD with 42–5% kcal from fat (De Taeye et al., 2010; Yang et al., 2021) compared to recent studies with HFD at 60% (Wang et al., 2017; Overby et al., 2020). Also, perhaps important for considering variability, the standard fat diets (standard chow) also vary depending on location and vendor from 12% (De Taeye et al., 2010), 13% (Lopez-Vicario et al., 2015), 14% (Yang et al., 2021), and 18% current studies. It is relevant in this scheme that the omega-3 enriched diet used here was a 15% fat diet that had equal amount of DHA and corn oil (omega-6 18:2), and thus is an enriched but at a standard fat level overall. The time interval of dietary intervention has varied from time intervals of 20 or 13 weeks (De Taeye et al., 2010) to 16 weeks (Lopez-Vicario et al., 2015) and 8 weeks (Wang et al., 2017; Overby et al., 2020). While the diet time interval has varied as widely as 5 weeks (Yang et al., 2021) to 5 months (Bettaieb et al., 2013), the studies have all been conducted in male animals. It is remarkable that none of these studies investigated female mice; thus, we have included them to elucidate changes due the difference in sex of the animals. Here, we also demonstrated the effect of diet in sEH knockout mice of both sexes.

### 4.1 sEH Activity

Increased sEH expression has been correlated with inflammatory pathology in the CNS (Ren et al., 2016; Hung et al., 2017) and cardiovascular system (Ai et al., 2009; Redina et al., 2017). In a previous study, significant change in sEH activity in adipose tissue was not observed. However, we note two important differences from this study compared to our current results. First, the HFD used in this study had a 42% kcal fat content compared to the 60% HFD used in several recent studies. Additionally, the lack of significant change in sEH enzyme activity in adipose tissue in male mice was observed in our current study as well; however, we did observe diet-induced change in male liver. Perhaps more impactful, we also observed significant change in sEH enzyme activity based on diet in the females.

Interestingly, the results demonstrated that LFD decreased sEH activity in the liver in WT females but showed no change on the HFD while the WT males responded to the HFD as well as n3FD with higher activity in the liver (Figure 1). The female WT mice appeared to be more affected by HFDs with increased sEH activity in BAT regardless of the class of fat (Figure 1C). Both the HFD and n3FD groups showed significantly increased activity in BAT compared to the lower fat content diets in WT females, and this was not true in the male WT mice. The fat content of the diets tended to increase activity in the WAT of both sexes but was limited and not significant likely given the variability for the small number of subjects. Remarkably, activity levels were low in both sexes compared to adipose tissues with the exception of the response to the n3FD. The n3FD significantly increased sEH activity in the WT females but not the males, though there was dynamic change for both sexes. Omega-3 fatty acids are preferentially taken up into the brain and it is possible that the sEH activity response in WT animals may correlate to subpopulations in the DHA transport system or even polymorphism in the sEH gene. There are perhaps many possible explanations for this variability, but they were not investigated with these experiments. The relationship of the n3FD diet to sEH activity in WT brain of both sexes did not result in substantially lower levels of the omega-3 metabolite EDPs in the brain of either sex. This effect was unanticipated and interesting that the variability occurred in both sexes; however, given the small number of individuals, it will need to be further explored.

The reduction in EpFA that is suggested to be due to lower expression of CYP enzymes (Wang et al., 2017) and increased she activity demonstrated here support the idea that inhibition of the sEH enzyme may be beneficial in controlling obesity. The EpFA have beneficial effects, which are limited due to their rapid degradation by sEH, which can their lower concentrations. The PUFA substrate availability to generate the EpFA, as well as the expression of both the phospholipases among other enzymes that liberate the substrates of sEH and the sEH expression itself remain unchanged (Wang et al., 2017). These observations illustrate that sEH inhibition may be the best approach for increasing the concentration of EpFA in these tissues to exert their beneficial effects. Inhibiting sEH is also beneficial by limiting diet-induced ER stress (Bettaieb et al., 2013), and decreasing diet-induced metabolic syndrome in obese rats (Iyer et al., 2012), renal injury in hypertensive obese rats (Imig et al., 2009), and endothelial disfunction in animal models (Zhang et al., 2011), among other biology.

The approach of sEH inhibition in obesity was investigated by Lopez-Vicario et al. (2015) using the fat-1 transgenic murine model. The fat-1 mice have a transgenic expression of a desaturase for omega-3 and enrich tissues with omega-3 PUFA. In this model using male mice, sEHI expanded brown adipose volume and limited ER stress as well as limiting hepatic steatosis. This suggested that sEH inhibition in combination with omega-3 enrichment is successful in countering the metabolic dysfunctions in obesity. This is supported by the data from our experiments that show that the effects of greatly increased EDP metabolites in sEH KO animals coincided with lower weight than WT animals on the same diet.

### 4.2 Body Weight

Overall, the body weights increased weight overtime as expected, with the greatest increased body weight on the HFD and a sexual dimorphism of males gaining more than females per genotype (Figure 2). As mentioned above, there was a noticeable effect of the diets between the WT and KO animals, which may indicate the role of sEH in weight gain and obesity in mice. It also supports data that showed the benefit of sEH inhibition to combat obesity. Interestingly, the WT male mice gained weight on the n3FD, which also correlated with higher sEH activity, though the diet itself has a lower percentage fat than the SFD.

Studies have suggested that sEH inhibition limits obesity and metabolic disease (Iyer et al., 2012; Xu et al., 2016) (Yang et al., 2021). In one such study, the administration of sEHI for 6 weeks did not alter male body weight once mice had been fed 8 weeks of HFD (Overby et al., 2020). Here, we demonstrate that sEH KO mice fed the diets for 8 weeks displayed less weight gain compared to WT mice of both sexes on HFD (Figure 2). Additionally, global knockout of the sEH enzyme was able to limit weight gain in the HFD without any weight loss or changes to average gain over time (8 weeks) on the lower-fat diets. Thus, longer-term inhibition of sEH may hold benefits for limiting obesity. Moreover, here the male KO mice had lower TG levels compared to WT males while the TC levels were not significantly affected (Figure 3), which is similar to previous reported uses of sEHI (Overby et al., 2020), (Yang et al., 2021). This could be of importance as lower triglycerides have been related to improved outcomes in vascular dementia and cognitive decline (Parthasarathy et al., 2017).

### 4.3 Oxylipins

#### 4.3.1 EpFA

In these experiments, we compared oxylipin levels with WT and the global sEH KO in both sexes. For the WT animals, the changes in liver oxylipins seemed to correlate with the sEH activity rather than appear as an inverse relationship. The WT females had lower sEH activity on LFD (Figure 1) as well as lower levels of EETs (Figure 4A). The WT males had higher sEH activity on HFD (Figure 1C) and had higher levels of EETs in liver (Figure 4A). EDPs also increased in liver of HFD fed WT males, which could occur due to the increased *α*-linolenic acid (precursor for DHA synthesis *in vivo*) in the soybean oil of this diet. This suggests that the overall dietary alteration affects more than sEH expression and the metabolism of EpFA. Interestingly, the previously investigated changes in gonadal adipose revealed changes of reduced cytochrome P450 gene expression (2J5, 2J6, and 2C44 isoforms) measured by PCR rather than a significant increase in sEH or phospholipase expression (Wang et al., 2017). Analysis of the fatty acid composition in this study demonstrated the gonadal fat pads consisted mostly of triglycerides, but the minimal levels of phospholipids present were not significantly altered by a 60% HFD. The authors concluded that the alteration of CYP expression was reduced and may have been impactful for EpFA levels, but change was due to a lack of PUFA substrates (Wang et al., 2017). Thus, the biosynthesis of EpFA may be impacted by diet as well. There are known sexual dimorphisms in both CYP450 expression, which mediates the biosynthesis of EpFA, and the expression of she, which degrades them. The results here with KO mice underscore the larger effects of sEH ablation compared to the WT sexual dimorphism in terms of EpFA levels. Other effects of diet were evident in liver with the n3FD reducing EETs in WT and sEH KO animals (Figure 4A) and greatly increasing EDPs in all animals (Figure 4A). However, more evident was the overall amount of oxylipin EpFA metabolites increasing in sEH KO mice compared to WT mice.

The scale of the differences between the WT and KO is remarkable and should be put into perspective. The global KO is of course more complete than a pharmacological intervention, but it does bring into perspective the advantages of small-molecule inhibition of sEH that balance the oxylipins in the desired manner. Overall, it was interesting to note that the most dynamic change in oxylipin concentrations was the amount of liver EDPs in female KO mice on the n3FD, which increased over all other diets in female KO, and was a greater absolute amount compared to male sEH KO and WT animals on all diets.

The BAT had overall lower levels of all depicted metabolites in both sexes and the knockout of sEH did not alter these levels as much as in other tissue types (Figure 4B). The change was most related to the fat type of the diet with the n3FD increasing EDPs and HFD increasing EETs levels in all mice more than genotype-related effects. In both WT females and males, the EET levels in BAT were robustly decreased on the n3FD. This compared to the effects in other adipose tissue where the fat content rather than type fat of the diet was more influential. In the WAT, the male WT EET levels were several-fold higher than WT females (note: scale differs between graphs) no matter which diet except the n3FD (Figure 4C). Also, there was a genotypic effect noticeable in the males with sEH KO levels higher in oxylipins compared to WT males. For the KO male compared to KO females, this was an even more striking increase in WAT EETs. The EDPs had a similar sex difference in WAT but with greater increases in EDPs on the n3FD comparatively. Interestingly, the females seemed to be influenced by the fat content of the diet with high levels of EETs for the WT female on HFD and high levels of EDPs for KO females on the n3FD.

The levels of EETs in brain were more comparable to the WAT for the WT males while the WT females had higher levels in brain than WAT (Figure 4D). An unexpected result was high levels of EET and EDP in the brain of LFD KO females. This seems to be an anomaly, but the methods were reviewed and in keeping with all other results. The WT males had very similar EET levels between the WAT and brain while the KO males had much lower EDP levels in brain than WAT.

The profound effect of sEH gene deletion on the EpFA is the most notable change and far exceed the changes due to sexual dimorphism within the genotype. Also remarkable are the effects of an omega-3 supplemented diet that exceeded the amount of change due to sexual dimorphism and was dynamic in lowering EET and increasing EDP metabolites in the liver in mice of both genetic backgrounds. Overall, the selected oxylipins were at the lowest levels in BAT and the BAT was the least affected by dietary change. The brain oxylipins were also not affected by dietary change but demonstrated more dynamic change based on sEH genotype with and also a sexual dimorphism among the sEH KO mice. The WAT oxylipin analysis demonstrated the largest sexual dimorphism in sEH KO mice overall. This of course indicates a mechanism that is in addition to the sexually dimorphic expression of sEH itself (Pinot et al., 1995) and should be explored in future experiments.

The relative success of inducing change in animals on HFD with sEH inhibitors has been previously described (Lopez-Vicario et al., 2015) and is not repeated here. Moreover, in support of previous data, our results demonstrate that there is increased sEH activity with higher fat content diets and that gene deletion can significantly increase EpFA metabolites. Small-molecule sEHI offer a potential to elicit similar effect as seen with gene deletion to preserve the EpFA allowing their beneficial actions. sEH inhibition has also been demonstrated to lower serum triglycerides (Overby et al., 2020), which is similar to results observed here in male mice where the sEH KO had lower TG than WT males. In our experiments, the liver triglycerides were significantly increased in HFD fed mice and liver TG levels have the potential to be lowered by sEHI as they were with the sEH knockout.

#### 4.3.2 Prostaglandins

Previous studies of prostaglandin (PG) oxylipins from WT male mice on HFD reported lower levels of PGE_2_ in gonadal WAT and BAT (Wang et al., 2017). Our study agreed with the PGE_2_ result in WAT from mice on HFD compared to SFD, but we observed an increased level of PGE_2_ in the BAT of HFD fed WT and sEH KO males (Figure 5B). Moreover, in liver, both WT males and females responded with increased PGE_2_ and PGD_2_ levels on the HFD. Thus, our study supports the hypothesis that HFD is able to increase PG metabolite levels. The PG levels in liver of the sEH KO animals did not increase equally on HFD, with higher PGD_2_ levels demonstrating a potential protective effect (Wang et al., 2021). The correlation of sEH ablation with lowering inflammatory metabolites relates to a recent finding using a 42% HFD where an sEHI in combination with select EpFA regioisomers limited NFΚB activation in BAT in mice on HFD (Yang et al., 2021).

The same 60% HFD used here was previously administered for a longer duration (5 and 10 months) to male mice, which both increased sEH protein and induced endoplasmic reticulum stress (ER stress) in the liver and adipose tissue (Bettaieb et al., 2013). In our study, we found that the WT male mice on HFD have an increase in sEH-specific activity in liver as well. Interestingly, the WT females on HFD did not exhibit a similar increase in sEH activity. On the longer-term study, the male KO mice demonstrated attenuated hepatic ER Stress on both SFD and HFD (Bettaieb et al., 2013). Both genetic ablation and sEHI limited ER stress in the adipose tissue in the male mice (Bettaieb et al., 2013). Although the sEH enzyme activity did not increase in HFD fed WT females, it is still possible that the sEH ablation or inhibition could decrease the ER stress markers in female mice; however, this remains to be determined in future studies.

## 5. Conclusion

These experiments demonstrated the role of sEH in regulating EpFA and its response to alterations in dietary lipids. Despite the small number comprising the treatment groups and the inherent individual variability, significant changes resulted from the diet interventions in both male and female mice of both WT and sEH KO genetic backgrounds. This demonstrates the critical role of sEH in the regulation of lipid mediators and correlates with several health outcomes that have been investigated in other studies. Here, the impact of not only sEH inhibition but also diet supplementation is evident and demonstrates the potential for impacting health. It is important to consider that studies are conducted in humans and that both sexes are included, and we have demonstrated not only in absolute amount but also in changes in portions of metabolites that there are differences in the metabolism of lipid mediators between females and males not only related to the sEH enzyme.

## Data Availability

The data presented in the study are deposited in the Dryad repository, and can be accessed via doi:10.5061/dryad.34tmpg4m4.
