# Supplementary material for: Soluble Epoxide Hydrolase Deletion Limits High-Fat Diet-Induced Inflammation
Source: Front Pharmacol. 2021 Dec 17;12:778470. doi: 10.3389/fphar.2021.778470 (PMC8719166; doi:10.3389/fphar.2021.778470)
Supplement: Supplementary file 1 [file DataSheet1.PDF]

Supplementary Material

Supplemental Figure 1. Mouse chow consumption over 8 weeks of diet treatment.
Wildtype (WT) or soluble epoxide hydrolase knockout (KO) mice of both sexes were fed one of the following diets for 8 weeks: high-fat diet, 60 kcal% fat (HFD), standard chow, 18kcal % fat (SFD), omega-3 DHA enriched diet 15 kcal% fat, 6.25% of omega-3 (n3FD), or low fat diet, 10 kcal% fat (LFD). **(A)** KO females responded to the HFD with a slight increase in chow consumption over other diets but stable consumption over time **(B)** KO males responded to the HFD with a slight decrease in chow consumption over other diets but stable consumption over time **(C)** WT females responded to the LFD and n3FD with lower consumption compared to HFD and SFD, but weight gain was most significant on the HFD (main text, Fig 2A) **(D)** WT males showed stable consumption but also had a significant weight gain on HFD (main text, Fig 2C).

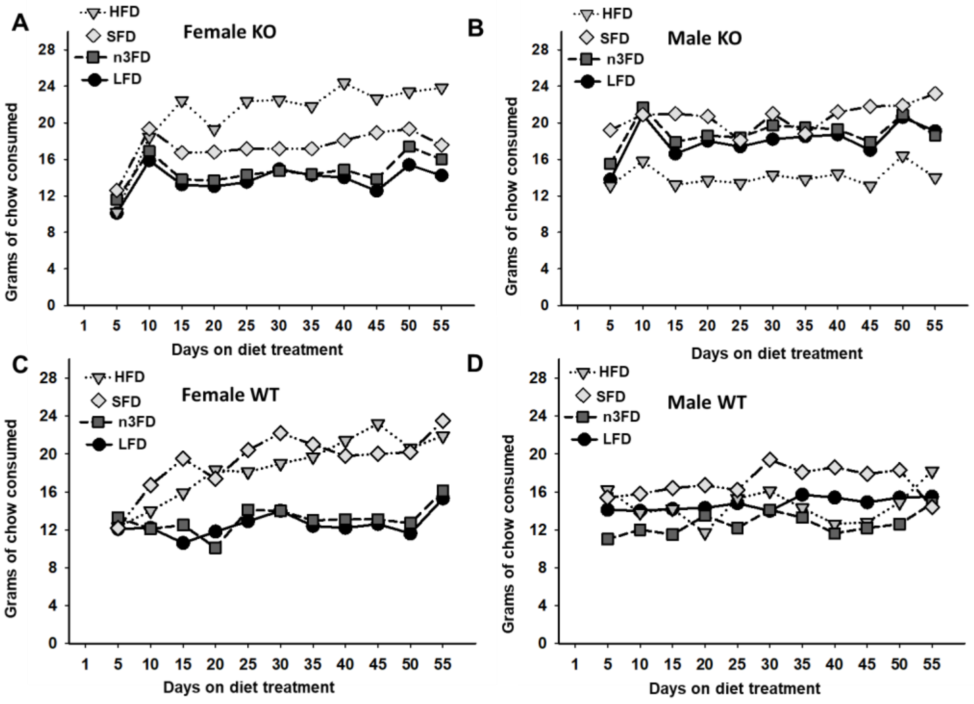

Figure S1

Supplementary Table 1. Soluble epoxide hydrolase activity in tissues.
The groups are reported per sex and genotype including wildtype (WT) C57/B6 and global soluble epoxide hydrolase knockout (KO) female (F) and male (M) mice grouped per the diet treatments including standard fat diet (SFD), low fat diet (LFD), high fat diet (HFD) and an omega-3 enriched diet (n3FD).

	**Specific Activity** nmol/min.mg/protein		**Total Activity** nmol/min.mg/tissue		**Specific Activity** nmol/min.mg/protein		**Total Activity** nmol/min.mg/tissue	
**Group**	**average**	**SD**	**average**	**SD**	**average**	**SD**	**average**	**SD**
	**LIVER**				**BAT**			
F WT SFD	29.9	2.66	9.25	1.74	4.48	0.51	0.238	0.040
F WT LFD	19.2	4.04	6.15	2.07	3.70	0.35	0.294	0.067
F WT HFD	32.1	4.23	7.31	3.37	7.02	0.57	0.274	0.121
F WT n3FD	34.1	3.16	7.39	1.48	5.67	0.58	0.553	0.327
M WT SFD	44.8	9.32	15.3	4.73	4.32	0.52	0.097	0.014
M WT LFD	48.2	3.62	12.5	1.55	4.56	0.46	0.154	0.037
M WT HFD	81.1	7.91	18.7	4.75	5.04	1.14	0.101	0.011
M WT n3FD	58.7	5.34	15.7	4.48	5.43	0.73	0.115	0.026
F KO SFD	0.029	0.012	0.012	0.003	0.021	0.009	0.001	0.001
F KO LFD	0.013	0.008	0.003	0.002	0.025	0.003	0.002	0.000
F KO HFD	0.032	0.009	0.012	0.007	0.027	0.005	0.001	0.000
F KO n3FD	0.023	0.011	0.005	0.002	0.016	0.002	0.001	0.000
M KO SFD	0.016	0.005	0.006	0.002	0.031	0.014	0.001	0.001
M KO LFD	0.023	0.015	0.005	0.004	0.016	0.005	0.001	0.000
M KO HFD	0.010	0.009	0.002	0.001	0.027	0.006	0.001	0.000
M KO n3FD	0.020	0.608	0.004	0.086	0.018	0.006	0.001	0.000
	**BRAIN**				**WAT**			
F WT SFD	1.68	0.31	0.026	0.002	1.70	1.05	0.009	0.002
F WT LFD	2.05	0.21	0.030	0.001	2.53	1.31	0.026	0.025
F WT HFD	1.93	0.19	0.030	0.001	3.73	0.70	0.006	0.007
F WT n3FD	8.07	7.39	0.052	0.027	2.64	1.57	0.026	0.023
M WT SFD	1.13	0.33	0.023	0.003	3.11	1.59	0.008	0.001
M WT LFD	1.20	0.19	0.026	0.003	2.76	0.78	0.012	0.008
M WT HFD	0.85	0.23	0.024	0.004	4.26	0.55	0.006	0.002
M WT n3FD	4.14	5.44	0.093	0.121	5.10	1.39	0.009	0.009
F KO SFD	0.039	0.006	0.001	0.000	0.140	0.062	0.002	0.001
F KO LFD	0.044	0.006	0.001	0.000	0.148	0.031	0.004	0.002
F KO HFD	0.043	0.007	0.001	0.000	0.270	0.065	0.001	0.001
F KO n3FD	0.031	0.003	0.001	0.000	0.142	0.024	0.002	0.000
M KO SFD	0.036	0.011	0.001	0.000	0.155	0.067	0.001	0.001
M KO LFD	0.025	0.006	0.001	0.000	0.086	0.008	0.002	0.002
M KO HFD	0.035	0.003	0.001	0.000	0.142	0.086	0.000	0.000
M KO n3FD	0.036	0.005	0.001	0.000	0.161	0.105	0.001	0.000

Supplementary Table 2. Oxylipin tissue concentrations.

The selected oxylipins are reported per sex and genotype including wildtype (WT) C57/B6 and global soluble epoxide hydrolase knockout (KO) female and male mice grouped per the diet treatments including standard fat diet (SFD), low fat diet (LFD), high fat diet (HFD) and an omega-3 enriched diet (n3FD). The quantification is reported as the average picomoles per gram tissue (pmol/g) ± SEM per group (n=3-4) of oxidized lipid metabolites. Select regioisomers of epoxy-fatty acids formed from arachidonic acid (epoxyeicosatrienoic acids, EETs) and docosahexaenoic acid (epoxydocosapentanoic acids, EDPs), their corresponding diol products dihydroxyeicosatrienoic acids (DHETs) and dihydroxydocosapentaenoic acids (DiHDPEs) respectively, and the linoleic epoxides (EpOMEs) and diols (DiHOMEs) are listed per tissue type. Finally, levels of select inflammatory prostaglandins (prostaglandin $E_{2,}$ PGE_2 and prostaglandin $D_{2,}$ $PGD_{2)}$ and lipoxygenase metabolites (LTB3, Leukotriene $B_{3;}$ LXA_4, Lipoxin $A_{4;}$ and Hydroxyeicosatetraenoic acids at the 5, 12 and 15 positions, 5-HETE, 12-HETE, 15-HETE) are included and listed per tissue type. The tissues included are liver, scapular brown adipose tissue (BAT), gonadal white adipose tissue (WAT), and brain.

LIVER

		EETs			EDPs			
		**14(15)**	**11(12)**	**8(9)**	**19(20)**	**16(17)**	**13(14)**	**10(11)**
**WT**	**SFD**	202± 69	262± 90	126± 41	83 ±29	54 ±20	54±21	80±30
**FEMALE**	**LFD**	144± 42	168± 50	95± 32	84±30	52±17	49 ±17	69± 22
	**HFD**	244± 59	322± 87	166± 45	137±18	91±20	89±21	129±31
	**N3FD**	44± 7	52± 9	25± 4	458±59	278±37	267±37	400±58
**SEH KO**	**SFD**	862 ±117	1306 ±241	628 ±149	515 ±58	415 ±85	396 ±74	575 ±103
**FEMALE**	**LFD**	885 ±206	1108 ±261	523 ±119	420 ±97	291 ±72	278 ±65	402 ±91
	**HFD**	1350 ±185	1950 ±52	939 ±35	772 ±56	581 ±30	550 ±30	785 ±42
	**N3FD**	185 ±39	216 ±48	99 ±22	2097 ±532	1405 ±364	1333 ±354	1926 ±523
**WT**	**SFD**	103 ±20	126 ± 26	61 ±11	50 ±5	32 ±4	31 ±3	43 ±4
**MALE**	**LFD**	75 ±14	96 ±19	49 ±10	35 ±6	25 ±5	24 ±5	36 ±7
	**HFD**	285 ±54	390 ±74	211 ±35	186 ±10	146 ±19	146 ±18	220 ±28
	**N3FD**	24 ±1	31 ±1	17 ±0.3	490 ±31	328 ±11	314 ±17	478 ±22
**KO**	**SFD**	811 ±155	1172 ±228	556 ±113	383 ±40	295 ±42	283 ±41	425 ±67
**MALE**	**LFD**	867 ±274	1219 ±401	609 ±217	449 ±140	338 ±111	326 ±107	495 ±171
	**HFD**	595 ±185	836 ±263	389 ±125	378 ±43	256 ±26	253 ±28	375 ±42
	**N3FD**	256 ±163	339 ±222	161 ±104	699 ±238	446 ±149	446 ±157	652 ±219

BAT

		EETs			EDPs			
		**14(15)**	**11(12)**	**8(9)**	**19(20)**	**16(17)**	**13(14)**	**10(11)**
**WT FEMALE**	**SFD**	1.2 ±0.9	2.3 ±1.3	2.3 ±1.4	2.1±0.9	0.3±0.2	0.5±0.3	1.1±0.8
	**LFD**	1.0 ±0.1	2.1 ±0.7	1.1 ±0.2	2.4±1.1	0.1±0.02	0.2±0.04	0.3±0.1
	**HFD**	1.4 ±0.2	1.4 ±0.1	1.4 ±0.2	3.4±2.3	0.3±0.1	0.3±0.04	0.4±0.1
	**N3FD**	0.5 ±0.1	0.5 ±0.1	1.6 ±1.3	18±4.3	1.9±0.4	1.8±0.3	2.7±0.5
**KO FEMALE**	**SFD**	5.3±1.0	4.7±0.5	3.1±0.3	4.5±1.8	0.8±0.1	0.8±0.1	1.2±0.2
	**LFD**	2.9±0.5	3.1±0.4	1.5±0.3	2.2±0.6	0.3±0.1	0.4±0.1	0.9±0.3
	**HFD**	3.2±0.3	3.2±0.4	1.5±0.2	1.0±0.1	0.3 ± 0.02	0.3±0.01	0.5 ± 0.02
	**N3FD**	1.5±0.3	1.5±0.4	0.6±0.2	31 ±6.0	5.6 ±1.8	4.4 ±1.5	7.0 ±2.6
**WT MALE**	**SFD**	1.3±0.2	1.0±0.2	0.4±0.1	1.2±0.6	0.1±0.04	0.2±0.1	0.3±0.1
	**LFD**	1.2±0.2	1.2±0.3	0.9±0.4	0.9±0.3	0.1±0.1	0.2±0.02	0.3±0.1
	**HFD**	2.0±0.3	2.5±0.7	2.5±0.8	1.6±0.3	0.3±0.1	0.5±0.1	0.8±0.2
	**N3FD**	0.4±0.1	0.6±0.1	0.3±0.1	11±2.0	1.8±0.3	1.7±0.4	2.9±0.7
**KO MALE**	**SFD**	4.7 ±1.0	5.6 ±0.7	2.6 ±0.6	2.2±0.4	0.7±0.1	0.7±0.1	0.9±0.2
	**LFD**	2.9 ±0.6	2.5 ±0.5	1.3 ±0.4	1.7±0.4	0.4±0.1	0.3±0.1	0.5±0.2
	**HFD**	5.3± 3.3	12 ±10	6.4 ±5.4	4.7±2.5	1.1±0.6	1.2±0.6	2.1±1.1
	**N3FD**	2.4 ±0.5	2.4 ±0.3	1.1±0.2	24±12	5.4±2.6	5.0±2.4	8.6±4.7

WAT

		EETs			EDPs			
		**14(15)**	**11(12)**	**8(9)**	**19(20)**	**16(17)**	**13(14)**	**10(11)**
**WT FEMALE**	**SFD**	1.9 ±0.6	1.5 ±0.3	0.6 ±0.1	1.1 ±0.2	0.4 ±0.1	0.4 ±0.1	0.4 ±0.1
	**LFD**	1.5 ±0.2	1.2 ±0.2	0.4 ±0.1	0.7 ±0.2	0.2 ±0.1	0.2 ±0.04	0.2 ±0.04
	**HFD**	31 ±11	22 ±10	7.0 ±3.6	8.3 ±2.9	4.5 ±1.6	4.0 ±1.5	4.0 ±1.8
	**N3FD**	0.9 ±0.1	0.6 ±0.1	0.1 ±0.03	15 ±1.1	5.5 ±0.5	4.4 ±0.5	4.1 ±0.6
**KO FEMALE**	**SFD**	4.2 ±0.7	2.4 ±0.5	1.4 ±0.3	6.0 ±2.7	0.5 ±0.1	0.5 ±0.1	0.65 ±0.1
	**LFD**	2.5 ±0.1	1.6 ±0.1	1.1 ±0.1	2.5 ±0.3	0.3 ±0.03	0.35 ±0.03	0.53 ±0.04
	**HFD**	2.5 ±0.2	1.5 ±0.1	0.5 ±0.04	1.3 ±0.2	0.24 ±0.03	0.4 ±0.1	0.33 ±0.1
	**N3FD**	3.1 ±1.7	1.5 ±0.8	0.7 ±0.3	49 ±23	6.2 ±3.6	4.8 ±2.9	5.6 ±2.3
**WT MALE**	**SFD**	91 ±32	117 ±42	48 ±18	30 ±11	18.7 ±7.1	19 ±7.3	26 ±9.6
	**LFD**	29 ±7	26 ±6.3	9.8 ±2.7	8.0 ±2.3	3.7 ±0.8	3.6 ±0.9	4.7 ±1.2
	**HFD**	49 ±9.6	47 ±10	18 ±3.8	8.9 ±1.5	4.5 ±0.7	4.5 ±0.9	6.1 ±1.1
	**N3FD**	11 ±3.1	12 ±3.3	4.1 ±1.1	94 ±18	51 ±10	47 ±9.7	65 ±15
**KO MALE**	**SFD**	104 ±64	153 ±100	70 ±46	50 ±33	34 ±23	32 ±22	48 ±32
	**LFD**	225 ±40	318 ±64	146 ±31	101 ±18	66 ±13	66 ±14	96 ±20
	**HFD**	126 ±33	192 ±55	85 ±25	108 ±44	69 ±27	68 ±27	94 ±36
	**N3FD**	99.6 ±36	149 ±67	66 ±31	317 ±161	214 ±111	207 ±105	310 ±157

BRAIN

		EETs			EDPs			
		**14(15)**	**11(12)**	**8(9)**	**19(20)**	**16(17)**	**13(14)**	**10(11)**
**WT FEMALE**	**SFD**	16 ±2.8	16 ±1.8	5.6 ±0.6	3.8 ±0.6	1.8 ±0.3	1.6 ±0.2	2.1 ±0.1
	**LFD**	21 ±10	21 ±8.4	7.6 ±2.8	5.1 ±2.2	2.7 ±1.2	2.4 ±1.1	3.2 ±1.3
	**HFD**	15 ±1.3	16 ±0.6	6.3 ±0.4	4.1 ±0.5	2.1 ±0.2	1.9 ±0.2	2.7 ±0.1
	**N3FD**	10 ±1.3	11 ±1	4.3 ±0.3	5.6 ±0.5	2.4 ±0.3	2.3 ±0.3	3.0 ±0.3
**KO FEMALE**	**SFD**	39 ±27	44 ±31	17 ±11	11 ±7.0	5.5 ±3.9	5.5 ±4.0	8.0 ±5.9
	**LFD**	289 ±141	288 ±142	96 ±48	63 ±30	38 ±19	37 ±18	49 ±24
	**HFD**	26 ±2.1	24 ±1.2	8.9 ±0.2	6.1 ±0.2	3.2 ±0.2	3.0 ±0.1	3.6 ±0.2
	**N3FD**	16 ±5.9	14 ±4.1	4.7 ±0.9	7.2 ±2.0	3.0 ±1.0	2.8 ±0.7	3.5 ±0.7
**WT MALE**	**SFD**	13 ±3.3	15 ±3.7	5.8 ±1.4	3.5 ±0.7	1.6 ±0.3	1.5±0.3	2.2 ±0.4
	**LFD**	8.7 ±1.3	8.8 ±1.1	5.4 ±1.0	3.1 ±0.6	1.1 ±0.2	1.1±0.1	1.6 ±0.2
	**HFD**	17 ±4.3	21 ±3.9	9.5 ±1	6.2 ±0.9	2.4±0.5	2.4 ±0.4	3.3 ±0.6
	**N3FD**	8.1 ±1.2	10 ±1.6	3.4 ±0.2	6.0 ±1.7	2.3 ±0.3	2.3 ±0.2	3.6 ±0.3
**KO MALE**	**SFD**	260 ±79	276 ±90	103 ±35	56 ±16	29 ±8.7	30 ±9.5	41 ±13
	**LFD**	105 ±36	123 ±43	47 ±16	24 ±7.7	14 ±4.8	13 ±4.7	18 ±6.6
	**HFD**	182 ±70	196 ±78	72 ±29	38 ±13	20 ±7.6	20 ±7.8	28 ±10
	**N3FD**	202 ±141	212 ±153	78 ±58	113 ±38	63 ±23	57 ±22	74 ± 33

LIVER

		DHETs			DiHDPEs			
		**14(15)**	**11(12)**	**8(9)**	**19(20)**	**16(17)**	**13(14)**	**10(11)**
**WT FEMALE**	**SFD**	30±8.7	29±8.0	17±4.8	42 ±15	12 ±4.3	10 ±3.2	11 ±3.6
	**LFD**	50±21	33±11	25±14	90 ±26	25 ±9.3	17 ±6.1	20 ±10
	**HFD**	72±10	57±12	52±13	114 ±29	33 ±5.6	24 ±5.3	34 ±8.6
	**N3FD**	12±1.5	7.9±1.2	4.8±0.8	389 ±52	93 ±12	48 ±6.7	56 ±8.0
**KO FEMALE**	**SFD**	18±3.1	15±2.7	7.8±1.9	35±6.6	7.9±1.3	4.6±1.0	4.8±1.1
	**LFD**	19±4.1	15±3.6	5.6±1.6	38±6.5	10±1.7	5.1±0.9	5.4±1.4
	**HFD**	20±1.7	14±2.3	5.3±0.9	61±7.5	14±2.1	5.3±1.0	4.1±0.6
	**N3FD**	4.8±0.7	4.6±1.1	2.5±0.5	82±23	22±7.1	15±4.9	16±4.1
**WT MALE**	**SFD**	35±3.8	17±1.2	11±1.0	58±6.8	11±0.9	5.7±0.7	6.1±0.7
	**LFD**	29±7.8	13±2.3	6.7±1.3	83±26	15±4.4	5.0±1.1	4.1±0.7
	**HFD**	116±11	52±22	46±24	684±180	113±6.2	44±22	57±42
	**N3FD**	17±2.1	4.3±0.7	3.4±0.9	946±18	178±14	54±11	60±18
**KO MALE**	**SFD**	18±4.1	14±1.5	5.6±1.1	27±2.2	6.7±0.6	3.2±0.4	2.6±0.3
	**LFD**	33±2.1	23±1.7	6.0±0.9	42±6.9	12±0.6	4.9±0.2	3.8±0.5
	**HFD**	10±3.7	7.1±2.0	2.6±0.9	32±5.6	7.3±1.4	2.5±0.5	2.1±0.4
	**N3FD**	6.5±2.7	5.6±2.1	1.8±0.7	65±24	13±4.7	8.0±3.0	6.8±3.3

BAT

		DHETs			DiHDPEs			
		**14(15)**	**11(12)**	**8(9)**	**19(20)**	**16(17)**	**13(14)**	**10(11)**
**WT FEMALE**	**SFD**	4.7±1.4	6.7±3.1	2.5±0.8	4.2±0.3	1.4±0.5	2.0±0.9	1.6±0.9
	**LFD**	4.2±0.8	3.5±0.3	2.0±0.2	7.9±2.8	1.2±0.2	1.0±0.1	0.8±0.1
	**HFD**	5.8±1.6	7.1±1.4	3.7±1.4	18±13.1	9.6±8.4	9.0±7.5	7.3±6.1
	**N3FD**	2.1±0.9	1.7±0.7	1.4±0.7	49±12	9.5±2.6	8.2±2.4	8.9±2.8
**KO FEMALE**	**SFD**	1.1±0.2	1.6±0.3	0.8±0.1	5.3±2.3	0.5±0.1	0.7±0.1	0.4±0.1
	**LFD**	1.4±0.2	1.5±0.2	0.7±0.1	4.5±0.5	0.6±0.1	0.6±0.1	0.4±0.1
	**HFD**	0.8±0.1	1.1±0.1	0.5±0.1	2.7±0.5	0.2±0.04	0.2±0.03	0.2±0.02
	**N3FD**	0.4±0.04	0.3±0.04	0.3±0.04	27±4.8	1.5±0.1	1.4±0.1	1.0±0.1
**WT MALE**	**SFD**	1.6±0.3	1.5±0.1	0.9±0.1	3.6±1.1	0.6±0.2	0.4±0.04	0.4±0.1
	**LFD**	2.7±0.6	3.8±1.4	1.5±0.5	3.5±0.4	0.8±0.2	0.9±0.2	0.6±0.2
	**HFD**	4.3±0.6	9.2±2.0	2.9±0.5	3.2±0.7	1.0±0.2	1.2±0.2	1.4±0.3
	**N3FD**	0.8±0.2	1.0±0.1	0.6±0.2	19±5.0	5.8±1.5	7.3±1.8	8.5±2.2
**KO MALE**	**SFD**	2.0±0.6	1.9±0.6	1.9±0.7	1.4±0.4	0.4±0.1	0.5±0.2	0.7±0.2
	**LFD**	0.8±0.2	1.0±0.3	0.5±0.03	3.5±0.9	0.3±0.1	0.3±0.1	0.2±0.1
	**HFD**	1.1±0.4	2.4±1.4	1.0±0.5	5.4±1.7	0.4±0.1	0.5±0.2	0.5±0.2
	**N3FD**	0.8±0.2	1.0±0.4	0.6±0.1	16±9.9	1.3±0.6	2.2±1.0	2.0±1.1

WAT

		DHETs			DiHDPEs			
		**14(15)**	**11(12)**	**8(9)**	**19(20)**	**16(17)**	**13(14)**	**10(11)**
**WT FEMALE**	**SFD**	0.2±0.03	0.1±0.02	0.2±0.03	5.1±1.9	0.5±0.1	0.3±0.1	0.4±0.1
	**LFD**	0.3±0.1	0.1±0.02	0.2±0.02	6.2±2.3	0.6±0.2	0.4±0.1	0.3±0.1
	**HFD**	0.2±0.1	0.1±0.05	0.2±0.1	2.0±0.7	0.4±0.1	0.3±0.1	0.3±0.1
	**N3FD**	2.7±0.6	0.8±0.1	1.0±0.3	52±12	4.9±0.9	2.5±0.5	3.4±0.5
**KO FEMALE**	**SFD**	0.2±0.02	0.2±0.03	0.2±0.02	2.9±0.8	0.3±0.05	0.2±0.04	0.2±0.1
	**LFD**	0.2±0.03	0.2±0.05	0.2±0.01	3.4±0.4	0.3±0.1	0.3±0.02	0.2±0.1
	**HFD**	0.1±0.01	0.1±0.01	0.1±0.02	1.2±0.3	0.2±0.03	0.1±0.03	0.1±0.04
	**N3FD**	1.7±1.2	1.1±0.6	1.2±0.8	40±31	1.6±1.1	2.1±1.6	2.1±1.5
**WT MALE**	**SFD**	0.2±0.05	0.1±0.02	0.3±0.1	3.1±0.8	0.3±0.1	0.2±0.04	0.2±0.1
	**LFD**	0.2±0.03	0.2±0.04	0.3±0.1	2.3±0.9	0.3±0.1	0.2±0.02	0.3±0.1
	**HFD**	0.1±0.001	0.1±0.005	0.3±0.029	0.6±0.1	0.1±0.01	0.1±0.005	0.1±0.01
	**N3FD**	0.7±0.1	0.3±0.03	0.3±0.04	9.6±2.0	1.1±0.3	0.8±0.3	1.1±0.3
**KO MALE**	**SFD**	0.1±0.02	0.1±0.02	0.2±0.05	1.8±0.6	0.3±0.1	0.4±0.1	0.3±0.1
	**LFD**	0.2±0.1	0.2±0.02	0.5±0.2	3.0±1.0	0.5±0.2	0.5±0.1	0.4±0.1
	**HFD**	0.2±0.1	0.2±0.03	0.4±0.1	3.4±0.6	0.4±0.2	0.3±0.1	0.4±0.2
	**N3FD**	2.2±1.4	1.7±1.0	1.5±0.8	16±10	2.5±1.7	2.4±1.6	3.0±1.7

BRAIN

		DHETs			DiHDPEs			
		**14(15)**	**11(12)**	**8(9)**	**19(20)**	**16(17)**	**13(14)**	**10(11)**
**WT FEMALE**	**SFD**	1.1±0.4	1.1±0.4	0.9±0.2	0.8±0.1	0.3±0.02	0.2±0.1	0.3±0.05
	**LFD**	0.9±0.1	1.0±0.1	0.9±0.1	0.8±0.2	0.2±0.03	0.2±0.03	0.3±0.03
	**HFD**	0.7±0.1	0.8±0.1	0.8±0.1	0.8±0.1	0.2±0.03	0.2±0.03	0.2±0.04
	**N3FD**	0.8±0.1	0.9±0.1	0.9±0.1	2.5±0.3	0.4±0.02	0.4±0.03	0.6±0.04
**KO FEMALE**	**SFD**	0.8±0.1	0.9±0.1	0.8±0.1	1.1±0.1	0.3±0.04	0.2±0.01	0.2±0.05
	**LFD**	1.0±0.3	0.9±0.2	0.8±0.2	1.1±0.2	03±0.1	0.2±0.1	03±0.1
	**HFD**	0.7±0.1	0.7±0.1	0.7±0.05	1.0±0.1	0.2±0.02	0.2±0.03	0.2±0.03
	**N3FD**	0.7±0.03	0.7±0.1	0.7±0.08	2.5±0.2	0.3±0.01	0.3±0.01	0.4±0.05
**WT MALE**	**SFD**	3.7±0.5	3.6±0.5	3.3±0.3	1.3±0.1	0.7±0.03	0.7±0.1	0.9±0.1
	**LFD**	6.3±0.7	5.8±0.8	5.0±0.8	1.3±0.1	1.1±0.1	1.1±0.2	1.5±0.2
	**HFD**	9.7±2.9	9.7±2.7	7.5±1.8	1.8±0.2	1.5±0.3	1.8±0.4	2.1±0.5
	**N3FD**	4.3±0.1	3.8±0.2	3.3±0.2	3.8±1.0	1.6±0.4	1.7±0.5	2.2±0.6
**KO MALE**	**SFD**	5.6±0.9	5.4±0.8	4.0±0.6	1.1±0.2	0.9±0.1	1.0±0.1	1.4±0.2
	**LFD**	2.5±0.1	2.6±0.3	1.8±0.2	1.0±0.2	0.6±0.1	0.6±0.1	0.7±0.1
	**HFD**	5.9±0.8	4.7±0.4	2.8±0.3	1.3±0.2	0.9±0.1	0.9±0.1	1.2±0.1
	**N3FD**	2.2±0.9	1.8±0.8	1.8±0.8	4.0±1.2	2.5±1.7	3.0±2.0	3.5±2.3

LIVER AND BAT

	**Liver**	EpOMEs		DiHOMEs		**BAT**	EpOMEs		DiHOMEs	
		**9(10)**	**12(13)**	**9(10)**	**12(13)**		**9(10)**	**12(13)**	**9(10)**	**12(13)**
**WT FEMALE**	**SFD**	1515±214	1502±217	246±65	423±103		16±6	16±4	115±39	196±27
	**LFD**	843±278	837±272	272±199	463±271		24±10	24±10	118±41	115±39
	**HFD**	1335±241	1413±223	627±185	788±154		8±1	9±1	69±19	70±18
	**N3FD**	746±105	722±102	144±30	333±49		17±3	19±4	74±24	81±26
**KO FEMALE**	**SFD**	5749±1529	5826±1535	60±12	203±51		73±23	127±49	36±5	36±4
	**LFD**	3095±336	3113±321	41±6	93±20		17±2	18±2	44±9	14±3
	**HFD**	5999±525	6282±465	38±1	72±2		21±3	24±3	15±2	5±1
	**N3FD**	2425±612	2146±495	20±6	41±14		43±20	47±19	10±1	6±1
**WT MALE**	**SFD**	724±85	737±82	112±27	386±66		35±28	37±29	97±44	245±116
	**LFD**	376±63	370±59	37±6	209±61		13±6	13±5	79±17	71±15
	**HFD**	1400±76	1326±52	436±349	890±135		10±2	9±1	64±18	62±16
	**N3FD**	625±14	575±15	103±30	553±73		15±4	13±4	106±37	100±35
**KO MALE**	**SFD**	4485±372	4535±360	35±2	242±73		4±2	12±8	11±5	23±8
	**LFD**	3123±1019	3069±1030	62±18	172±41		22±5	29±6	46±12	11±2
	**HFD**	2688±775	2766±807	22±9	59±19		56±41	51±31	37±15	13±4
	**N3FD**	2041±547	1965±548	36±10	66±14		64±29	62±23	54±21	20±7

WAT AND BRAIN

	WAT	EpOMEs		DiHOMEs		Brain	EpOMEs		DiHOMEs	
		9(10)	12(13)	9(10)	12(13)		9(10)	12(13)	9(10)	12(13)
WT FEMALE	SFD	14±2	18±2	49±7	148±38		5±2	10±5	2±0.7	4±1
	LFD	12±2	15±2	55±21	66±24		1±1	2±1	1±0.001	1±0.3
	HFD	421±258	533±314	18±5	25±9		4±1	5±2	1±0.5	2±1
	N3FD	11±2	14±2	24±3	36±5		1±0.2	2±0.3	0.2±0.01	1±0.1
KO FEMALE	SFD	88±42	183±66	37±5	37±10		5±3	8±3	0.3±0.2	2.5±0.8
	LFD	19±2	19±2	29±2	9±1		47±23	53±26	1.1±0.4	1.3±0.6
	HFD	10±2	14±2	11±1	3±0		7±2	8±2	0.8±0.4	1.6±0.3
	N3FD	42±14	52±18	34±26	30±24		1±0.4	2±0.4	0	1.5±0.6
WT MALE	SFD	2229±987	2413±1081	46±11	177±53		1±1	5±2	1±1	5±2
	LFD	400±103	448±115	29±4	39±5		2±1	2±1	2±1	2±1
	HFD	556±110	630±121	7±1	9±1		5±2	6±2	5±2	6±2
	N3FD	721±104	806±110	16±5	21±3		5±4	6±4	5±4	6±4
KO MALE	SFD	1314±639	1334±617	43±8	52±18		39±6	44±7	2±0.2	4±1
	LFD	2173±713	2176±685	50±16	60±21		12±5	13±5	0.5±0.2	1±0.3
	HFD	1698±2914	3847±1873	61±20	49±19		83±19	85±23	76±50	119±80
	N3FD	2914±1284	2873±1271	119±75	138±101		39±12	49±15	7±6	9±6

PROSTAGLANDINS

		Liver		BAT		WAT		Brain	
		PGE_2	PGD_2	PGE_2	PGD_2	PGE_2	PGD_2	PGE_2	PGD_2
WT FEMALE	SFD	9.2±2.7	7.1±3.0	3.0±1.7	2.1±0.8	1.2±0.4	1.5±0.5	4.0±0.8	27±5.0
	LFD	5.2±3.5	2.9±1.4	1.6±0.5	2.4±1.3	1.5±0.6	1.7±0.6	3.7±0.3	29±0.9
	HFD	22±4.9	16±3.0	1.9±0.7	1.9±1.0	2.5±1.0	1.1±0.3	3.3±0.9	24±7.1
	N3FD	2.0±1.5	1.7±0.4	0.6±0.4	0.9±0.4	0.4±0.1	0.5±0.1	3.3±0.2	22±1.3
KO FEMALE	SFD	2.4±0.7	2.9±0.7	2.7±1.0	1.6±0.4	8.0±2.5	5.6±2.0	3.9±0.7	29±5.5
	LFD	3.7±1.4	6.9±4.4	1.1±0.3	0.7±0.2	4.8±0.5	3.4±0.4	3.2±0.4	21±1.5
	HFD	1.9±0.6	2.4±0.7	1.2±0.5	1.0±0.2	2.2±0.2	1.8±0.1	3.5±0.3	29±1.0
	N3FD	2.1±1.5	0.9±0.3	0.1±0.04	0.4±0.1	2.4±1.9	1.8±1.0	2.1±0.5	14±3.7
WT MALE	SFD	2.2±0.6	3.8±1.5	1.7±0.3	1.2±0.2	11±4.2	4.3±1.5	4.3±0.3	24±2.4
	LFD	0.9±0.2	1.8±0.4	0.9±0.4	0.7±0.2	2.5±1.4	2.1±0.5	6.5±1.9	24±1.2
	HFD	10±6.7	12±5.3	6.9±2.6	3.5±1.1	3.7±1.5	1.8±0.3	7.2±1.2	30±2.9
	N3FD	0.8±0.4	1.0±0.5	0.6±0.2	0.5±0.1	1.5±1.1	0.9±0.4	3.2±0.3	15±1.3
KO MALE	SFD	1.9±0.3	3.0±06	5.3±3.7	27±7.8	20±10	5.9±2.1	3.9±0.6	18±3.6
	LFD	5.9±3.2	7.0±2.5	1.0±0.1	1.1±0.1	4.2±1.2	7.8±2.2	2.4±0.3	14±1.9
	HFD	1.5±0.6	4.2±1.3	8.1±7.8	3.8±3.3	11±11	5.9±5.1	5.1±1.1	18±2.2
	N3FD	1.0±0.3	2.0±0.8	0.7±0.4	0.8±0.1	7.8±3.8	5.4±1.9	2.5±0.2	11±5.6

LIVER

		LTB3	LXA4	5-HETE	12-HETE	15-HETE
**WT FEMALE**	**SFD**	0.6±0.2	17±9	139±20	324±156	444±121
	**LFD**	1±1	9±9	222±135	326±97	654±375
	**HFD**	1±1	42±13	338±84	431±129	994±254
	**N3FD**	0.2±0.1	12±3	45±12	88±21	165±48
**KO FEMALE**	**SFD**	1±0.1	4±1	88±13	165±20	240±37
	**LFD**	1±0.3	4±1	98±3	246±65	277±21
	**HFD**	1±0.2	4±0.3	171±46	289±33	324±60
	**N3FD**	2±2	19±16	41±20	47±20	120±59
**WT MALE**	**SFD**	0.2±0.1	2±1	76±7	212±4	283±48
	**LFD**	0.2±0.0	0.6± 0.1	47±13	121±23	137±31
	**HFD**	1±0.1	21±17	254±110	200±77	494±221
	**N3FD**	0.2±0.1	7±2	30±8	37±9	73±20
**KO MALE**	**SFD**	0.6± 0.1	4±1	58±7	104±14	153±25
	**LFD**	0.4± 0.1	4±1	87± 36	198±76	209±48
	**HFD**	0.4± 0.1	2±1	30±11	54±22	88±29
	**N3FD**	0.6± 0.3	6±3	27±8	75±25	88±23

BAT

		LTB3	LXA4	5-HETE	12-HETE	15-HETE
**WT FEMALE**	**SFD**	0.2±0.1	2±1	48±14	264±82	193±57
	**LFD**	0.03±0.01	1±0.2	40±10	193±30	106±14
	**HFD**	0.1±0.04	7±5	51±15	140±17	145±26
	**N3FD**	0.1±0.02	4±2	28±11	738±664	88±37
**KO FEMALE**	**SFD**	0.04±0.01	0.3± .1	21±3	336±83	89±16
	**LFD**	0.03±0.003	0.5±0.2	26±6	157±32	12±23
	**HFD**	0.1 0.01	0.2±0.03	11±1	162±52	59±4
	**N3FD**	0.03±0.01	0.5±0.1	8±1	63±10	29±2
**WT MALE**	**SFD**	0.2±0.005	0.3±0.1	13±3	47±7	74±20
	**LFD**	0.03±0.01	0.4±0.2	23±7	85±26	121±59
	**HFD**	0.5±0.4	4±1.3	54±11	263±34	200±41
	**N3FD**	0.02±0.01	5±1.2	7±1	29±2	25±1
**KO MALE**	**SFD**	0.1±0.03	1±0.4	36±13.2	159±47	265±116
	**LFD**	0.02±0.003	0.2 0.05	14±0.4	265±116	53±1
	**HFD**	1±1	6±5	61±57	53±1	232±206
	**N3FD**	0.1±0.1	1±1	12±2	232±206	57±14

WAT

		LTB3	LXA4	5-HETE	12-HETE	15-HETE
**WT FEMALE**	**SFD**	0±0	0.3±0.1	4±2	147±23	33±7
	**LFD**	0.004±0.003	0.1±0.02	4±1	150±26	24±4
	**HFD**	0.01±0.004	0.4±0.1	6±2	51±16	20±9
	**N3FD**	0.002±0.001	0.2±0.03	1±0.3	31±10	8±1
**KO FEMALE**	**SFD**	0.004±0.003	0.1±0.02	6±1	329±113	46±14
	**LFD**	0.01±0.01	0.2±0.1	8±2	326±78	71±21
	**HFD**	0.002±0.002	0.1±0.04	3± 0.6	127 ±43	21±5
	**N3FD**	0.01±0.01	0.7±0.6	10± 8	165± 82	40±25
**WT MALE**	**SFD**	0.1±0.03	0.6±0.1	3±1	109±33	22±7
	**LFD**	0.6±1	1±1	1±1	134±51	16±6
	**HFD**	0.1±0.02	4±1	12±1	31±8	25±5
	**N3FD**	0.2±0.1	17±13	3±2	17±4	11±4
**KO MALE**	**SFD**	0.1±0.1	1.9±1	6.1±1	107±32	30±9
	**LFD**	0.1±0.1	0.8±0.1	6.1±1	198±40	35±6
	**HFD**	0.2±0.1	3.6±3	9.7±5	52±27	45±2
	**N3FD**	0.1±0.04	1.5±1	5.4±1	79±35	23±8

BRAIN

		LTB3	LXA4	5-HETE	12-HETE	15-HETE
**WT FEMALE**	**SFD**	0.03±0.03	0.4±0.02	24±3.2	117±20	112±19
	**LFD**	0.004±0.004	0.2±0.03	27±1.3	141±13	121±13
	**HFD**	0.01± 0.002	0.2±0.1	20±2.6	101±21	90±16
	**N3FD**	0.01±0.01	0.1±0.04	21±1	88±6	99±8
**KO FEMALE**	**SFD**	0.02±0.01	0.3±0.01	25±4	198±45	124±14
	**LFD**	0.01±0.004	0.3±0.1	22±6	164±38	99±18
	**HFD**	0.02±0.01	0.3±0.03	23±1	254±18	102±2
	**N3FD**	0.01±0.004	0.3±0.05	29±4	94±26	128±23
**WT MALE**	**SFD**	0.01±0.002	0.3±0.1	36±5	234±43	204±23
	**LFD**	0.01±0.004	0.6±0.1	111±15	213±63	290±49
	**HFD**	0.03±0.01	1.2±0.5	104±34	445±92	533±160
	**N3FD**	0.01±0.01	0.5± 0.1	68±14	269±21	183±29
**KO MALE**	**SFD**	0.04±0.02	1.1±0.5	81±22	170±43	328±93
	**LFD**	0.002±0.002	0.2±0.03	26±3	132±11	114±16
	**HFD**	0.03±0.01	2.8±1.8	83±24	242±36	361±109
	**N3FD**	0.03±0.01	0.8±0.3	21±9	82±34	100.5±42
